# Chemometric Classification of Cocoa Bean Shells Based on Their Polyphenolic Profile Determined by RP-HPLC-PDA Analysis and Spectrophotometric Assays

**DOI:** 10.3390/antiox10101533

**Published:** 2021-09-27

**Authors:** Olga Rojo-Poveda, Giuseppe Zeppa, Ilario Ferrocino, Caroline Stévigny, Letricia Barbosa-Pereira

**Affiliations:** 1 Department of Agriculture, Forestry and Food Sciences (DISAFA), University of Turin, 10095 Grugliasco, Italy; giuseppe.zeppa@unito.it (G.Z.); ilario.ferrocino@unito.it (I.F.); letricia.barbosa.pereira@usc.es (L.B.-P.); 2 RD3 Department-Unit of Pharmacognosy, Bioanalysis and Drug Discovery, Faculty of Pharmacy, Université libre de Bruxelles, 1050 Brussels, Belgium; Caroline.Stevigny@ulb.be; 3 Department of Analytical Chemistry, Nutrition and Food Science, Faculty of Pharmacy, University of Santiago de Compostela, 15782 Santiago de Compostela, Spain

**Keywords:** cocoa bean shell, cocoa byproduct, RP-HPLC-PDA, spectrophotometric assays, radical scavenging activity, principal component analysis, polyphenols, methylxanthines, fingerprints, cocoa markers

## Abstract

The cocoa bean shell (CBS), a byproduct from the cocoa industry, was recently proposed as a functional and low-cost ingredient, mainly because of its content in polyphenols. However, vegetal food products could significantly differ in their chemical composition depending on different factors such as their geographical provenience. This work is aimed to determine the polyphenolic and methylxanthine profile of different CBS samples and utilize it for achieving their differentiation according to their geographical origin and variety. RP-HPLC-PDA was used to determine the CBS polyphenolic profile. Spectrophotometric assays were used to obtain the total phenolic, flavonoid, and tannin contents, as well as to evaluate their radical scavenging activity. The results obtained from both methods were then compared and used for the CBS differentiation according to their origin and varieties through chemometric analysis. RP-HPLC-PDA allowed to determine 25 polyphenolic compounds, as well as the methylxanthines theobromine and caffeine. Polyphenolic profile results highlighted significant differences among the analyzed samples, allowing for their differentiation based on their geographical provenience. Similar results were achieved with the results of the spectrophotometric assays, considered as screening methods. Differentiation based on CBS variety was instead obtained based on the HPLC-determined methylxanthine profile.

## 1. Introduction

The cocoa bean shell (CBS) is one of the main byproducts of the chocolate manufacturing industry, usually discarded after cocoa bean husking. In contrast to other cocoa byproducts such as the pod and the mucilage, CBS is usually discarded in a later step of the cocoa manufacture process, and it was reported that more than 700,000 tons of CBS waste are produced worldwide annually [1]. This creates added costs in the cocoa manufacturing process, mainly linked to the added weight that CBS represents during cocoa bean transportation and the environmental and economic cost of its disposal or underutilization. 

CBS represents about 10–17% of the total cocoa bean weight and was reported to be rich in proteins, dietary fibers, minerals, vitamins, and, most importantly, methylxanthines and polyphenols [1]. Theobromine and caffeine are two well-known methylxanthines present in cocoa and cocoa-derivative products that were reported to be in part responsible for the high acceptance of cocoa among consumers [2]. This is related to the capacity of these molecules to act in the central nervous system, influencing alertness and mood in a positive way. Besides the neurostimulating properties, caffeine and theobromine were also reported to possess several other beneficial properties for human health, such as anticarcinogenic, diuretic, and antiobesity effects, among others [2]. Also, cocoa beans and their derivative products are rich in polyphenols, concretely flavanols [3]. These compounds are secondary metabolites of plants with several benefits for human health when consumed as food [4]. They display antioxidant properties and were demonstrated to act as chemopreventers against several diseases such as cancer, diabetes, oxidative stress, cardiovascular diseases, and inflammatory bowel diseases, among others [5,6,7,8,9]. For this reason, and in the frame of a circular economy for a zero-waste cocoa industry, CBS was lately proposed as a low-cost ingredient for functional foods [10], mainly baked goods [11] and beverages [12,13]. Besides, its aromatic profile was previously reported to be very similar to that of cocoa powder [14], and it could therefore be an interesting ingredient with which produce cocoa-based products with a lower fat content and higher amounts of dietary fibers [1].

However, as a vegetable product, CBS chemical composition can significantly differ based on several factors, such as their geographical provenience or plant genotype, and the globalization of the cocoa industry could lead to issues concerning cocoa products’ traceability. Concretely, geographic origin was reported to be one of the main authenticity issues for food products in Europe [15]. Increasing attention was given to sustainability in cocoa production, for which geographical traceability becomes mandatory. Improving this aspect could ensure transparency along the supply chain and ensures cocoa and CBS sustainability, quality, and safety [16]. In recent years, chemometric techniques implying multivariate analyses arose to assure food quality and authenticity, as well as to classify their geographical origin or genotype based on their chemical composition. Some of these techniques were applied to cocoa beans [17]. However, being a novel ingredient, studies concerning CBS traceability are limited in the literature [14,18]. Henceforth, since food authentication depends on measuring features that can discriminate foods of different origins [15], establishing fingerprints based on the concentration of key compounds in CBS could help in the estimation of the byproduct traceability and quality in cases in which the purpose is to revalorize it as a functional food ingredient.

As CBS attracts the interest of food researchers and industries, studies concerning its polyphenolic content appeared in the literature in recent years. Nevertheless, most of these studies imply one or few similar CBS samples or aim at a few of the already known and representative compounds in CBS. The aim of the present study was to cover this gap by providing broader information about the polyphenolic profile and methylxanthine content in CBS, implying a large number of samples that could allow for extensive characterization of the cocoa byproduct. Additionally, spectrophotometric assays were performed, and the feasibility of these methods as polyphenolic content determination screening methods was assessed. In a second step, the results obtained with both methods were employed for a chemometric classification of the CBS samples according to their geographical origin and cocoa variety. 

## 2. Materials and Methods

### 2.1. Chemicals and Standars

Folin&Ciocalteu’s phenol reagent, sodium carbonate (≥99.5%), 2,2′-diphenyl-1-picryl hydrazyl (95%; DPPH), 6-hydroxy-2,5,7,8-tetramethylchroman-2-carboxylic acid (97%; trolox), vanillin (99%), (+)-catechin hydrate (>98%), methanol (≥99.9%), hydrochloric acid (37%), aluminum chloride (99%), sodium nitrite (≥99%), formic acid (≥98%), quercetin-3-O-glucoside (≥90%; Q-3-G), theobromine (≥98.5%), caffeine (≥98.5%), *p*-coumaric acid (≥98.0%) and quercetin (≥98.5%) were provided by Sigma–Aldrich (Milan, Italy). Gallic acid, ethanol (≥99.9%), sodium hydroxide (1 M), (-)-epicatechin (≥99%), procyanidin B1 (≥98.5%; PCB1), and protocatechuic acid (>97%) were supplied by Fluka (Milan, Italy).

Ultrapure water was prepared in a Milli-Q filter system (Millipore, Milan, Italy).

### 2.2. Samples

A set of 88 CBS samples were yielded from fermented and dried cocoa beans (*Theobroma cacao* L.) harvested during the season 2014/2015. The cocoa beans, purchased from several local cocoa manufacturers, came from 19 different countries in both continents America (*n* = 28) and Africa (*n* = 16) and belonged to four cocoa varieties: Forastero (*n* = 21), Trinitario (*n* = 15), Criollo (*n* = 6), and the Nacional variety of Ecuador (*n* = 2) as presented in Table 1. Due to confidentiality issues of the suppliers, information about the fermentation and drying conditions of the samples is not available. However, to standardize samples, a similar roasting process was performed for all the cocoa beans at 130 °C (isothermal) during 20 min on a ventilated Memmert UFE 550 oven (ENCO, Spinea, Italy), prior to the unshelling process. After manual separation from the beans, CBS samples were ground into a 250μm-particle size powder by using an ultracentrifugal mill Retsch ZM 200 (RetschGmbh, Haan, Germany) and stored under vacuum at −20 °C in no-light environment until extraction and analysis.

### 2.3. Polyphenolic Extracts

Extraction of polyphenols and methylxanthines was performed by mixing 0.50 g of cocoa bean shell powder with 10 mL of ethanol-water mixture (50:50, *v*/*v*) for 2h at 25 °C under constant rotatory oscillation (3 oscillation/s) using a VRL 711 orbital shaker (AsalS.r.l., Milan, Italy), according to the optimized extraction conditions for CBS described by Barbosa–Pereira et al. [19]. The mix was then centrifuged on a centrifuge Heraeus Megafuge 11R (THERMO electron, Chateau–Gontier, France) at 4200× *g* for 10 min at 4 °C before collecting and filtering the supernatant through a 0.45 μm PTFE filter. Extractions were performed in duplicate for each CBS sample.

### 2.4. Spectrophotometric Assays

#### 2.4.1. Total Phenolic, Flavonoid, and Tannin Contents

Three spectrophotometric screening methods were employed for rapid polyphenolic quantification depending on the different characteristics of these molecules. The total phenolic content (TPC), total flavonoid content (TFC), and total tannin content (TTC) were assessed by the Folin–Ciocalteu, aluminum chloride, and vanillin methods, respectively. Methods were adapted as described in [19] to be performed in 96-well microplates, and a BioTek Synergy HT spectrophotometric multidetection microplate reader (BioTek Instruments, Milan, Italy) was used. Analyses were performed in triplicate, and final concentrations were expressed according to different calibration curves. For the TPC, the obtained values were expressed as mg of gallic acid equivalents per gram of CBS (mg GAE/g CBS), according to a calibration curve drawn from 20 mg/L to 100 mg/L. For both TFC and TTC, catechin was used to build the standard curve (5–500 mg/L), and concentrations were expressed as mg of catechin equivalents per gram of CBS (mg GAE/g CBS).

#### 2.4.2. Antioxidant Capacity

The antioxidant capacity of the CBS polyphenolic extracts was determined according to the DPPH radical scavenging method described by [19]. The antioxidant capacity of the CBS extracts was calculated as the DPPH radical inhibition percentage as follows: $\mathrm{IP}\;\left(\%\right)=\frac{\left(A_{0}-A_{30}\right)}{A_{0}}\times 100$, where A_0_ is the initial absorbance and A_30_ is the absorbance after 30 min. Analyses were performed in triplicate and the concentrations values were based on a Trolox standard curve (12.5–300 μM) and expressed as micromoles of Trolox equivalents per gram of CBS (µmol TE/g CBS).

### 2.5. RP-HPLC-PDA Analysis

Detection and quantification of polyphenolic molecules and methylxanthines contained in the CBS extracts were performed by reversed-phase high-pressure liquid chromatography with a photodiode array detector (RP-HPLC-PDA) Thermo–Finnigan Spectra System (Thermo–Finnigan, Waltham, MA, USA). The instrument was composed by a P2000 binary gradient pump, SCM 1000 degasser, AS 3000 automatic injector, and Finnigan Surveyor PDA Plus detector. Data were acquired, analyzed, and processed by using the ChromQuest software 5.0 (Thermo–Finnigan, Waltham, MA, USA).

A reverse-phase Kinetex Phenyl-Hexyl column (150 × 4.6 mm, particle size of 5 μm) (Phenomenex, Castel Maggiore, Italy) was used at 35 °C to obtain the compound separation.

Water with 0.1% *v*/*v* formic acid and methanol served as mobile phases (solvent A and B, respectively). The sample injection volume was 10 μL and the gradient elution method described by [13] was applied with a flow rate of 1 mL/min during 45 min as follows: 0–2 min, 90% A and 10% B; 2–18 min, linear gradient from 10% to 50% B; 18–40 min, linear gradient from 50% to 80% B; 40–42 min, linear gradient from 80% to 90% B; and minutes 42–45, linear gradient until 90% A and 10% B.

Compound detection was performed with a continuous wavelength scanning between 200 and 400 nm. Quantification data for each compound were acquired at its maximum and more representative wavelength, indicated in Table 2.

Compound identification was based on standards injection and on a previous work performed in our laboratory in which mass spectrometry was used coupled to HPLC as molecular identification method [20].

For quantification, different external calibration curves that ranged from 0.2–100 mg/L of standard were analyzed under the same conditions, obtaining in all the cases correlation coefficients higher than R^2^ = 0.9980. Results were expressed as g/kg of CBS for methylxanthines (theobromine and caffeine) and as mg/kg of CBS for polyphenols.

### 2.6. Statistical and Chemometric Analyses

Chemometric analysis was performed based on the data obtained for the 88 CBS samples (Table 1). The 88 CBS samples were extracted and analyzed in triplicate. A principal component analysis (PCA) was applied based on the polyphenolic compounds’s normalized data (log_10_). The package *made4* of RStudio (https://www.r-project.org, accessed on 23 September 2021) and the *dudi.pca* function were used to discriminate the groups of samples by geographic area of origin or different cultivars based on their polyphenolic compounds. Analysis of similarity with 999 permutations was carried out with the anosim function of the *vegan* package of R with the aim of finding significant differences depending on the sample origin or cultivar. Kruskal–Wallis and Wilcoxon nonparametric tests were used to identify specific compounds or groups differentially abundant between origins or cultivars. Data for the specific compounds or compound groups driving the separations were represented by means of Box-plot graphics, which show the concentration range comprised between the first and third quartiles, with error bands that highlight the lowest and the highest values. Bonferroni’s correction for multiple comparisons was applied; a false discovery rate (FDR) of 0.05 or lower was considered statistically significant.

## 3. Results and Discussion

The present work was subdivided into two main parts. On the first step, the polyphenolic profile of the CBS samples was determined, following two different approaches. Different spectrophotometric assays (total phenolic content, total flavonoid content, total tannin content, and antioxidant capacity) were used as screening methods, while HPLC analysis was used as a complete characterization method. On the second step of this work, principal component analyses (PCA) were applied to the obtained results for a chemometric differentiation of the CBS samples according to their geographical origin or variety based on their polyphenol and methylxanthine contents.

### 3.1. Polyphenolic Quantification by Spectrophotometric Analyses

Spectrophotometric analysis of the phenolic content in CBS were performed in this study as screening methods, with the aim of comparing their results to those obtained by RP-HPLC-PDA analysis. Concretely, the total phenolic content (TPC), total flavonoid content (TFC), total tannin content (TTC), and the radical scavenging activity (RSA) were assessed through the Folin–Ciocalteu, aluminum chloride, vanillin, and DPPH radical scavenging methods, respectively. The results obtained for these for parameters for each CBS sample are shown in Table 3.

TPC values among the different samples ranged from 6.10 (UGA2) to 42.97 (PER2) mg GAE/g CBS. For cocoa beans, Criollo variety usually presents lower polyphenolic contents that account for 2/3 of what is found in Forastero variety beans [3]. Trinitario variety is a consequence of the botanical cross of the two previously mentioned varieties and should therefore have TPC values among those two. Nacional variety is close in aroma to the Criollo and Trinitario varieties and is therefore considered as the third ‘fine aroma’ variety. Nevertheless, from a botanical point of view it actually belongs to the Forastero group [21], although it is a native variety to Ecuador, and it should therefore contain TPC values close to the ones found for Forastero CBS. However, when looking into our sample set results, those differences and variety-dependence of the TPC values are not observed, and values seem to vary independently of the CBS variety. A clear example of this can be observed in the fact that the sample with the lowest TPC value (UGA2) is a Forastero CBS, while the one with the highest value (PER2) belongs to the Trinitario variety. On the contrary, a tendency depending on the CBS origin continent can be observed in which American samples tend to show higher TPC values than those of African ones. This general line can be observed with the fact that the lowest TPC values were found for samples coming from Uganda and Ghana (UGA1, UGA2, and GHA), while the highest ones were found for CBS samples coming for Mexico or Peru (PER2 and MEX). This trend can be explained by the different climatic conditions in both continents and the different cultivation traditions that lead to different chemical transformations because of the different fermentation and drying processes employed by the local populations. In our work, cocoa bean roasting and husking processes for yielding the different CBS were standardized as it could were done in the cocoa manufacturing industry. However, fermentation and drying processes that normally take place at the cocoa farm are hard or impossible to standardize when it comes to samples with different origins. Indeed, regulations applied in EU countries where cocoa is manufactured are not applied in the beginning of the supply chain (including fermentation and drying of cocoa beans) [16], which explains the common absence of exchanged information concerning the first treatments of cocoa raw material and, therefore, the impossibility of standardizing these first steps in the manufacturing process. Polyphenol content in cocoa beans and CBS gets reduced during fermentation since cocoa polyphenols can diffuse into fermentation sweating during the process. Nonstandardized fermentations could led to 6-fold variations in the final product polyphenol concentration [3]. Previous studies reported values of CBS TPC varying for 2.72 to 55.16 mg GAE/g CBS [13,19,22,23,24,25], which is in line with the ones obtained in the present work and confirms that variation in these parameters is mainly linked to the different origins, cultivation techniques, and fermentation and drying processes. When it comes to cocoa beans, previous studies found TPC values of 30.49–94.33 mg GAE/g [17,26,27]. TPC values in cocoa beans get to double those found for CBS, which is in line with what observed for individual polyphenolic compounds in the next section.

A comparison was made between the obtained TPC values (considered as a screening method) and the sum of the polyphenolic compound concentration obtained by HPLC to compare both methods. In general, it was observed that TPC values were an order of magnitude higher than the values obtained when adding all the individual compound concentrations (Table 3). One of the reasons explaining this difference could be the fact that there is probably a large amount of CBS polyphenols that remain undetermined and were not quantified in our study, although the most abundant and known compounds were taken into account. Spectrophotometric analyses are rapid and easy methods that can be performed by less experienced personnel than that which HPLC requires. However, these methods demonstrated to present several interferences that usually result in overestimated values. These are valid methods for screening and comparison between similar samples. However, chromatographic techniques are recommended to accompany these methods and establish a structure-activity relationship [28]. Nevertheless, a correlation coefficient of *r* = 0.8671 was found among the obtained TPC values and the HPLC-determined sum of concentrations, which confirms that spectrophotometric assays remain useful as approximate methods for semiquantitative phenolic amount determination and comparison among the different CBS samples. This correlation was much higher than the *r* = 0.32 found for the sum of individual phenols with TPC reported for cocoa beans [26].

TFC accounted for a 22.51–52.12% of the TPC. These numbers are in line with the 25.6% that the sum of flavan-3-ol, catechin-3-O-glycosides, flavonols, and flavonol-3-O-glucosides represented in the total polyphenolic compound concentration determined by RP-HPLC-PDA (Table 3). On the other hand, TFC values correlated with the flavonoid sum with a correlation coefficient of *r* = 0.8264. TFC values were comprised between 1.42 (UGA2) and 19.67 (SAT3) mg CE/g CBS. These values are slightly lower than previously found TFC values for CBS such as those in the work of Barbosa–Pereira et al. [19], in which TFC values of 5.39–43.94 mg CE/g CBS were reported. Nevertheless, in that work, a pulsed electric field assisted extraction was performed, which possibly influenced a bigger CBS flavonoid extraction over the rest of phenolic compounds. Although TFC values seemed to be overestimated as observed before for TPC, these values were highly correlated with the TPC obtained results (*r* = 0.9607), as observed before [13,19,29,30].

TTC results accounted for a 15.33–40.00% of the TPC values, with values ranging from 1.14 (UGA2) to 11.00 (PER2) mg CE/g CBS and were correlated with this parameter with *r* = 0.9251. The sum of all the procyanidins detected and quantified by HPLC (Table 3), which are considered as condensed tannins [3], accounted for a 51.0% of the total polyphenols quantified by HPLC, which is higher than the percentage range of TTC with respect to TPC, but still in line with those numbers. TTC values correlated with the values of HPLC-determined procyanidins with *r* = 0.7716. These compounds are believed to increase their concentration during the cocoa bean fermentation process when polyphenols diffuse from the storage cells and undergo oxidation to condensed tannins [3]. TTC values therefore highly depend on cocoa fermentation conditions and time. In previous works concerning CBS, TTC was reported to be comprised between 7.24 and 25.79 mg CE/g CBS when performing a pulsed electric field assisted extraction [19]. As happened for TFC, this kind of extraction performed in the mentioned work could have favored a major tannin extraction from CBS.

Polyphenols, and concretely cocoa and CBS polyphenols were reported to possess antioxidative properties that, together with other biofunctionalities, can help to treat or prevent several chronic diseases such as cancer, diabetes, and cardiovascular diseases, among others [1,5,6,7]. In the present study, the antioxidant characteristics of the CBS samples were measured as their radical scavenging activity (RSA), and the results gave values comprised between 27.69 (UGA2) and 181.98 (PER2) µmol TE/g CBS. The lowest and highest RSA values were obtained for the same samples that gave the lowest and highest values of TPC, TFC, and TTC. Indeed, RSA values were highly correlated with those three parameters with the following correlation coefficients: *r*_(RSA-TPC)_ = 0.9871, *r*_(RSA-TFC)_ = 0.9771, and *r*_(RSA-TTC)_ = 0.9480. These values are higher than those reported by Gültekin–Özgüven et al. [29] (*r*_(RSA-TPC)_ = 0.7010 and *r*_(RSA-TFC)_ = 0.7725), and those reported by Barbosa–Pereira et al. [19] (*r*_(RSA-TPC)_ = 0.9647, *r*_(RSA-TFC)_ = 0.9740, and *r*_(RSA-TTC)_ = 0.7290). Besides, the correlations of RSA with the HPLC-determined counterparts of the spectrophotometric parameters were *r*_(RSA-Total sum of polyphenols)_ = 0.8721, *r*_(RSA-Sum of flavonoids)_ = 0.8165, and *r*_(RSA-Sum of procyanidins)_ = 0.8446. Concerning the potential of the different HPLC-determined groups to act as antioxidant compounds, it was found that the highest correlations with RSA were found for PCA glycosides and PCB trimers (*r*_(RSA-PCAglycosides)_ = 0.8221 and *r*_(RSA-PCBtrimers)_ = 0.8100, respectively). Other works in which CBS antioxidant activity was studied reported values of 36–321.97 µmol TE/g CBS when employing assisted extractions [19,31]. These values are again higher than the ones reported in the present study, but they are logical if we take into account the also higher values of TPC, TFC, and TTC that were found in those works, as previously mentioned. Concerning cocoa beans, they were reported to show an antioxidant activity of 173 µmol TE/g roasted cocoa beans [29], which is in the range of the values found for CBS in the present work. Therefore, CBS, which is considered a byproduct, shows similar antioxidant capacity to that of cocoa nibs.

### 3.2. Polyphenolic and Methylxanthine Profile of the CBS Characterized by RP-HPLC-PDA 

The polyphenolic compounds and methylxanthines identified and quantified by RP-HPLC-PDA are shown in Table 2 and the chromatograms of a representative CBS extract and by RP- HPLC-PDA are shown in Figure S1 of the Supplementary Materials.

In Table 2, for each polyphenolic compound or methylxanthine, the peak number within the total chromatogram, retention time, and maximum wavelength at which the compound was quantified are indicated. The range of the different concentrations obtained for all of the different CBS extracts in our sample set for each compound is shown and expressed as milligram per kilogram of CBS. A total of 27 compounds comprising polyphenols and methylxanthines were detected and quantified. These compounds were grouped in different categories for which the total quantities (sum of the compound concentrations) are shown in Table 3 for each CBS sample. Table S1 of the Supplementary Materials shows an extended vision of Table 3, with the concentration for each individual compound within the different group categories.

The compounds with the most notable concentrations throughout the CBS samples were theobromine and caffeine, followed by flavanol monomers and procyanidins, similarly to what was previously reported for cocoa beans [32]. Theobromine concentrations in CBS ranged from 7.65 (CAM1) to 9.03 (DOR1) g/kg of CBS, while caffeine values were comprised between 0.15 (GHA) and 3.37 (VEN7) g/kg CBS. These values are in accordance with the ranges of 3.9–18.3 g of theobromine per kg of CBS and 0.4–4.2 g of caffeine per kg of CBS, which were reported in other studies gathered in the work of Rojo–Poveda [1]. In cocoa beans or cocoa powder from cocoa nibs, the concentrations of both methylxanthines were reported to be 1–24.28 g of theobromine per kg of cocoa powder and 0.5–7.28 g of caffeine per kg of cocoa beans, depending on the variety, origin, and fermentation, drying or roasting conditions [17,26,27,33,34,35]. Thus, contrary to some other chemical compounds such as polyphenols, which are generally more abundant in cocoa beans than in CBS, theobromine and caffeine concentrations found in the cocoa byproduct can be considered almost equivalent to those found in cocoa powder, although to a lower extent in the case of caffeine. Methylxanthines are believed to be synthesized in the cocoa cotyledons. However, during the fermentation process, there is a considerable migration of caffeine and theobromine towards the shell [1]. Therefore, concentrations of these compounds vary mostly depending on the fermentation state of cocoa beans. Studies focusing on CBS generally use fermented samples which were obtained from cocoa manufacturers in an advance step of the cocoa manufacturing process, whereas studies focusing on cocoa beans use both nonfermented (directly obtained from the plantations) and fermented and dried samples. This could be the reason why methylxanthine concentration ranges vary more in cocoa bean samples when compared to that of CBS samples.

The total amount of polyphenols quantified by RP-HPLC-PDA ranges from 119.96 mg/kg CBS (GHA) to 3403.22 mg/kg CBS (SAT2). Throughout the whole sample set, a total of 25 individual phenolic compounds were detected, identified, and quantified in the CBS samples. These individual compounds were further classified into nine different categories (Table S1): phenolic acids, flavan-3-ols, catechin-3-O-glycosides, procyanidins B-type (PCB), procyanidins B-type trimers (PCB trimers), procyanidins A-type (PCA) glycosides, flavonols, flavonol-3-O-glycosides, and *N*-phenylpropenoyl-L-aminoacids. The range of concentration among the CBS samples for each compound is shown in Table 2, while the sums of the concentrations of all the individual compounds forming each category for each CBS sample are shown in Table 3.

Protocatechuic acid was the only phenolic acid detected in CBS in the present study, although other studies reported the presence of other phenolic acids in CBS such as gallic acid (up to 147 mg/kg) [36], homovanillic acid, vanillic acid glycoside isomers, and cinnamic acid derivatives [22]. In our work, protocatechuic acid represented on average the 7.0% of the total quantified phenolic compounds, and its concentrations ranged from 11.89 mg/kg CBS (BRA) to 241.01 mg/kg CBS (MEX). Protocatechuic is a known phenolic acid contained in cocoa beans, and it was found in concentrations comprised between 2.20 and 22.2 mg/kg of cocoa bean [32], which is considerably lower than the concentrations found in CBS. Nevertheless, Cádiz–Gurrea et al. [22] claimed that CBS samples are characterized by a higher content in phenolic acids when compared to that of cocoa beans, while the latter have in general higher flavonoid contents.

Flavan-3-ols was one of the most abundant polyphenolic groups in the CBS samples, with concentrations that ranged from 17.19 (GHA) to 887.71 (MAD) mg/kg CBS and amounted on average the 20.0% of the total analyzed compounds, which is in agreement with the 20.5% of abundance found for the same compound group in cocoa beans by Rodríguez–Carrasco et al. [37]. Indeed, this group was formed by catechin and epicatechin, which are two of the most representative polyphenolic compounds in cocoa beans and CBS [1,3]. In our samples, catechin was present in concentrations that varied from 12.72 (GHA) to 180.37 (ECU7) mg/kg CBS. Other researchers that performed different types of optimized extractions with CBS such as the application of high-voltage electric discharge [36,38] and pressurized liquid extraction [31] found concentrations up to 290, 284, and 178 mg of catechin per kg of CBS, respectively, which is in accordance with the amounts quantified in this work. When it comes to cocoa beans, values of catechin ranging from 40.1 to 1297 mg/kg are reported in the literature [17,32,34], which is not far from what is observed for CBS. However, as stated before, these concentrations vary and decrease with the fermentation process [3], which happens to be more variable when in it refers to cocoa bean studies. Epicatechin was in general the most abundant individual polyphenolic compound found in our samples, and its concentration varied from 4.47 (GHA) to 748.79 (MAD) mg/kg CBS. Epicatechin was found in concentrations ranging from 210 up to 3500 mg/kg CBS in the previously mentioned works in which assisted extractions were performed [19,31,36,38], which is still in line with the values found in the present study. Regarding the concentrations of epicatechin in cocoa beans that are reported in the literature, these are slightly higher than those found in the cocoa byproducts, with values that vary from 500 to 4787 mg of epicatechin per kg of cocoa bean [17,32,34,35], although some studies reported concentrations up to 8510 mg of epicatechin per kg of cocoa bean [27]. However, it is worth mention that in this latter work, fermented but not roasted cocoa beans were studied, so a further decrease in epicatechin concentration due to the high roasting temperature did not take place.

Catechin-3-O-glucoside represented in average 2.3% of the total amount of quantified phenolic compounds, with a concentration range of 6.40 (UGA1)–101.49 (SAT2) mg/kg CBS. Catechin-3-O-glucoside was previously detected in aqueous extractions of CBS, prepared as beverages [12,13]. 

Procyanidin B-type (dimer) was, after epicatechin, the most abundant individual polyphenolic compound in CBS. It represented 12.9% of the total amount of quantified polyphenols and was present at concentrations of 6.84 (GHA)–672.55 (VEN2) mg/kg CBS. Seven different procyanidin B-type trimers were detected and quantified in almost all the CBS samples. Only PCB trimer 6 was absent in ECU2 and PCB trimer 2 was absent for UGA1. The sum of the seven PCB trimers was comprised between 26.28 (GHA) and 653.73 (SAT2) mg/kg CBS and accounted on average for 19.8% of the total quantified polyphenols. On the other hand, five glycosides of procyanidins A-type (PCA) were detected and quantified, among which PCA hexoside 1 was the most abundant. The sums of the PCA glycosides ranged from 16.34 (GHA) to 833.22 (SAT2) mg/kg CBS, and this compound group accounted for 18.3% of the total amount of polyphenols. Procyanidins are, together with their monomers (catechin and epicatechin), cocoa polyphenol markers [3]. In cocoa powder, procyanidin dimers were detected in concentrations of 1820–2710 mg/kg while trimers were detected in concentrations of 500–1200 mg/kg [34]. As observed before for flavanol monomers, procyanidin concentrations in CBS could be considered an order of magnitude below those found in cocoa nibs. This fact was also observed by Cádiz–Gurrea et al. [22], who stated that higher flavonoid contents were present in bean samples, while a higher content of phenolic acids was observed in CBS samples. However, it was found that flavanol monomers and procyanidins (including glycosides) in CBS accounted for 73.2% of the total quantified phenolic compounds, which is in line with the 60% stated for cocoa beans by Barnaba et al. [32].

Concerning flavonols, quercetin and two quercetin glycosides (quercetin-3-O-glucoside, also named isoquercitrin, and quercetin-3-O-arabinoside) were detected and quantified throughout the CBS sample set. Quercetin was found in concentrations going from 1.07 (IVC) to 22.37 (MEX) mg/kg CBS, while isoquercitrin concentrations ranged from 3.47 (GHA) to 47.59 (ECU3) mg/kg CBS and quercetin-3-O-arabinoside was present in concentrations comprised between 3.76 (GHA) and 86.53 (SAT2) mg/kg CBS. These compounds were claimed to increased their contents in the outer plant of the cocoa fruit to protect the plant when it is submitted to intense sunlight and irrigation [21], which could be related with their different concentrations in the different CBS samples. Quercetin accounted only for 0.3% of the total quantified polyphenols, while its glycosides were 3.1% of it. In CBS, flavonols were, therefore, present in much lower concentrations compared to flavanols, as it was already noticed for cocoa beans [3]. Nevertheless, the three flavonol compounds quantified in this study are considered to be characteristic from cocoa products and, therefore, cocoa markers [39]. In previous studies involving CBS, both quercetin and isoquercitrin were detected, together with other quercetin glycosides such as quercetin-3-O-galactoside or quercetin-3-O-rhamnoside [12,13,40]. In cocoa powder, these compounds were found in slightly higher concentrations when compared to that of the cocoa byproduct studied here. Quercetin-3-O-glucoside (isoquercitrin) was found in concentrations of 117–323 mg/kg of cocoa powder while quercetin-3-O-arabinoside was detected in concentrations of 180–486 mg/kg of cocoa powder [34].

*N*-phenylpropenoyl-L-aminoacids are hydroxycinnamate derivatives unique to cocoa products and are, therefore, cocoa markers [41]. The presence of these compounds was related to the astringent properties of cocoa [22], which are also very perceptible in CBS [13]. In this study, five amino acid derivatives were identified and quantified: *N*-coumaroyl-L-aspartate (isomers 1 and 2), *N*-caffeoyl-L-aspartate, *N*-coumaroyl-L-glutamate, and *N*-coumaroyl-L-tyrosine. Among the CBS samples, the sums of their concentrations were comprised between 17.13 (GHA) and 810.01 (MEX) mg/kg CBS, and they accounted on average for a 16.4% of the total quantified polyphenolic compounds. The most abundant compound in this category was *N*-caffeoyl-L-aspartate, which showed concentrations up to 652.58 mg/kg CBS (MEX). This amino acid derivative was also found to be the most abundant in cocoa beans [34,41]. In fermented cocoa beans, the sum of *N*-phenylpropenoyl-L-aminoacids was found to be 1470 mg/kg, an order of magnitude higher than that found in CBS [42].

### 3.3. Classification of CBS Based on Their Polyphenolic Fingerprints and Spectrophotometric Measurements

#### 3.3.1. Classification of CBS Samples According to Their Geographical Origin

PCA analyses of the dataset were performed to evaluate sample separation according to their geographic origin or variety. Polyphenolic amounts in cocoa were reported to be dependent on the cocoa cultivar [3]. However, in our study, when comparing varieties from America and Africa, no differences were observed since no tendencies with respect to the CBS variety were a priori found (see Section 3.1 and Section 3.2). 

**Figure 1:**
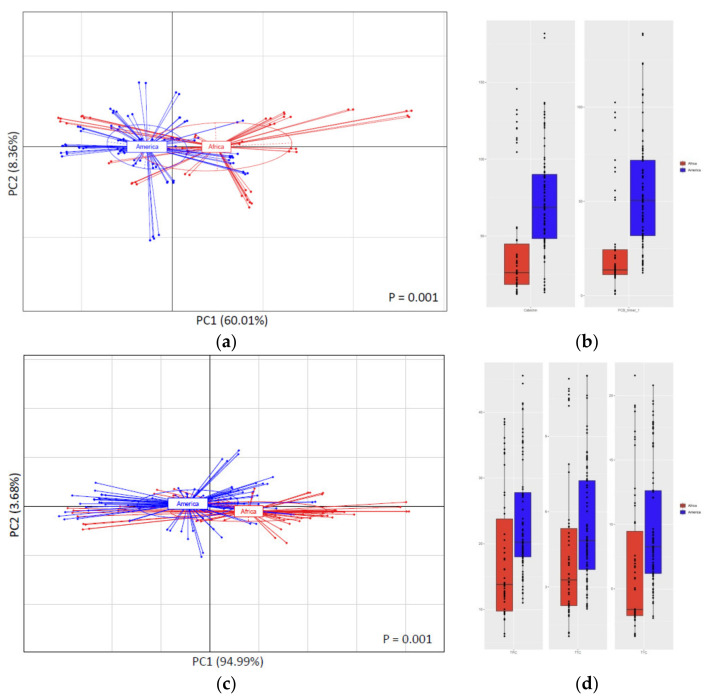
Principal component analysis (PCA) based on HPLC-determined polyphenolic profile (**a**) and spectrophotometric analyses results (**c**) as function of continent of origin of the CBS. Variance explained by first and second components of PCA is shown in parenthesis for PC1 and PC2 in each graph. Boxplots showing abundance of two key polyphenolic compounds (catechin and PCB trimer 1) (**b**) and total phenolic content (TPC), total tannin content (TTC), and total flavonoid content (TFC) (**d**) that can be used as possible markers for separation based on continent of origin are shown next to their correspondent PCA graph. For interpretation of legends, see Table 1.

On the other hand, a clear separation among American and African samples was found when performing the PCA as a function of origin (Figure 1). These PCAs explained 68.42% and 98.67% of the variance for the HPLC and spectrophotometric analyses plots, respectively. The separation was significant for both techniques (*p* = 0.001) and occurred in a similar way. The American samples appeared clustered at the left-side of the PCA diagram, while the African samples were placed at the right-side of the PCA diagram for both techniques (HPLC and spectrophotometric assays, Figure 1a,c, respectively).

Thus, the screening methods allowed for a similar separation of samples than the more sophisticated characterization method (HPLC). This could be enormously interesting from the cocoa industry point of view in terms of quick, easy, and cheap origin authentication of CBS samples. Origin classification based on the carbohydrate composition of fermented and unfermented cocoa beans was previously sought. However, this differentiation could not be clearly obtained and indicators to discern cocoa beans depending on a specific origin could not be proposed [43]. In another study involving different cocoa metabolites, a differentiation among African an Asian cocoa beans was attempted, although American samples seemed to cluster together with both groups [16]. Regarding the cocoa byproduct, attempts to chemometrically classify CBS samples were already reported. Mandrile et al. [18] worked on the authentication of CBS by infrared spectroscopy and optical emission spectroscopy, observing that the combination of those techniques allowed to individualize some patterns related to the geographical origin. Barbosa–Pereira et al. [14,44] found that it was possible to separate the Criollo variety from the rest of varieties according to the CBS volatile profile. On the contrary, in this work, the variety separation was not possible according to the polyphenolic profile, whereas the geographic separation was observed. As mentioned in previous sections, CBS polyphenolic profile seemed to be more dependent on the different factors linked to the cultivation areas (cultivation traditions, different climate conditions and different ecophysiological conditions) than the CBS varieties, a fact that was previously reported also for cocoa beans [27].

A deeper analysis into the origin classification allowed to observe that 24 out of the 25 HPLC-determined polyphenolic compounds had a significantly different content in CBS samples (FDR < 0.001 (12 compounds), FDR < 0.01 (9 compounds), and FDR < 0.05 (1 compound)), as shown in Table S2 of the Supplementary Materials. Catechin (FDR = 3.4 × 10^−8^) and PCB timer 1 (FDR = 3.3 × 10^−11^) (Figure 1b), among others, can be considered as markers in the discrimination of CBS. For the spectrophotometric analysis (Figure 1d), higher values for the different parameters were observed for the American continent in all cases. A similar tendency was found in a previous work involving cocoa beans [32], in which catechin was found to be more abundant in American cocoa beans than in African cocoa beans, and was also selected as a marker for the geographical separation of cocoa bean samples.

**Figure 2:**
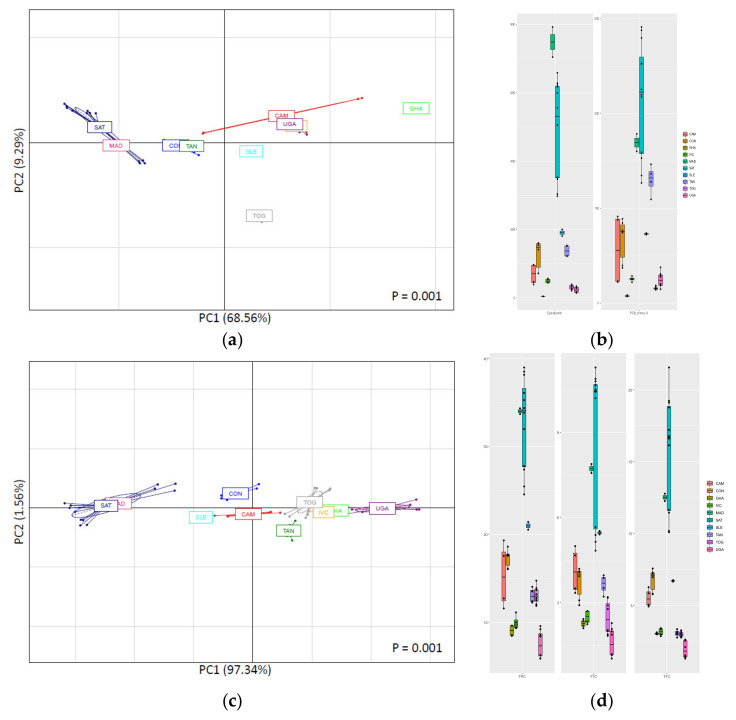
PCA based on HPLC-determined polyphenolic profile (**a**) and spectrophotometric analyses results (**c**) as function of country of origin among African CBS. Variance explained by first and second components of PCA is shown in parenthesis for PC1 and PC2 in each graph. Boxplots showing abundance of two key polyphenolic compounds (epicatechin and PCB trimer 4) (**b**) and TPC, TTC, and TFC (**d**) that can be used as possible markers for separation based in country of origin are shown next to their correspondent PCA graph. For interpretation of legends, see Table 1.

Figure 2 shows the PCA plots obtained for the African CBS samples, classified as function of the different countries of origin. 

These PCAs explained 77.85% of the total variance for the HPLC technique (Figure 2a) and 98.90% for the spectrophotometric analysis (Figure 2c). Both PCAs gave significant separation of the samples (*p* = 0.001) based on the country of origin, while the PCAs of the same samples and analytical techniques depending on the CBS variety showed no significant separations.

In both PCA plots shown in Figure 2a,c, the most notable characteristic was the clusterization of Madagascar and São Tomé CBS, which was well-differentiated from the other countries. The results obtained for the screening method (spectrophotometric assays) were similar to those obtained with the HPLC results. Two main compounds that drove the separation in the HPLC-results PCA plot were epicatechin and PCB trimer 4 (Figure 2b and Table S3). Epicatechin and PCB trimer 4 were found at their highest concentrations in Madagascar and São Tomé CBS. These two islands (characterized by high temperatures and humidity) share specific climatic conditions that make CBS polyphenol content similar. These environmental conditions were reported to enrich the concentrations of catechins, low-molecular weight flavan-3-ol oligomers, and total extractable procyanidins in cocoa [17]. Concerning TPC, TTC, and TFC (Figure 2d), Madagascar and São Tomé CBS gave the highest concentrations for all the three parameters, which drove the sample separation. As reported in Section 3.1, UGA samples were those with the lowest values in TPC, TTC, and TFC. This fact can also be observed in the plots of Figure 2d and was also the driving cause for the differentiation of the Uganda samples at the right-side of the spectrophotometric assay PCA plot.

**Figure 3:**
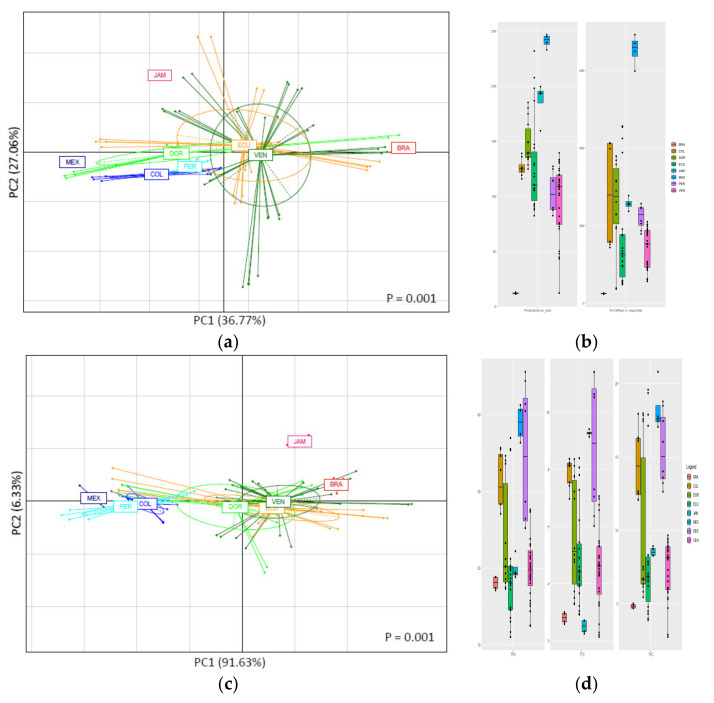
PCA based on HPLC-determined polyphenolic profile (**a**) and spectrophotometric analyses results (**c**) as function of country of origin among American CBS. Variance explained by first and second components of PCA is shown in parenthesis for PC1 and PC2 in each graph. Boxplots showing abundance of two key polyphenolic compounds (protocatechuic acid and *N*-caffeoyl-L-aspartate) (**b**) and TPC, TTC and TFC (**d**) that can be used as possible markers for separation based in country of origin are shown next to their correspondent PCA graph. For interpretation of legends, see Table 1.

Figure 3 shows the PCA plots obtained for the CBS samples from the American countries. These PCAs explained 63.83% of the total variance for the HPLC technique (Figure 3a) and 97.96% for the spectrophotometric analyses (Figure 3c). Similar to what was observed for African samples, PCA plots for the separation of the American samples based on the country of origin were found to be significant (*p* = 0.001), while those based on the CBS variety gave no significant separations.

HPLC results showed the separation of Mexican, Jamaican, and Brazilian samples from the rest of the samples. All the HPLC-determined polyphenolic compounds (except for PCA pentoside 1) contributed significantly to the differentiation of samples among the American countries (Table S4). Protocatechuic acid and N-caffeoyl-L-aspartate were found to be those with the highest significant differences among samples and can be used as markers for the separation based on American countries (Figure 3b). Mexican samples showed to be those with the highest concentrations of both markers, while Brazilian samples were those with the lowest concentrations. Jamaican samples were shown to possess high amounts of protocatechuic acid, but intermediate concentrations of N-caffeoyl-L-aspartate, while the rest of the samples showed contents of both compounds that varied proportionally. This, together with variations in concentrations of other compounds found in Table S4, could be the reason for the differentiation of Jamaican CBS samples. For the PCA of the spectrophotometric results, the CBS samples from Mexico, Jamaica, and Brazil were again differentiated from the others. Mexican samples were different because of their highest TPC, TFC, and TTC values, while the Brazilian ones showed the lowest values (Figure 3d). On the other hand, Jamaican samples were probably separated because of their unusually low values of TTC in comparison with their TPC and TFC values.

#### 3.3.2. Classification of CBS According to the Cultivar Localized Case Study (Ecuador)

**Figure 4:**
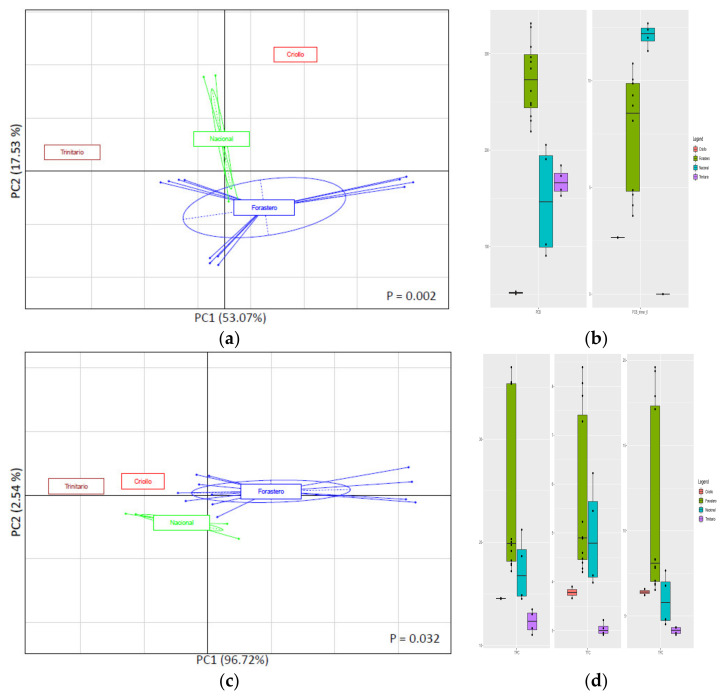
PCA based on HPLC-determined polyphenolic profile (**a**) and spectrophotometric analyses results (**c**) as function of CBS variety among the Ecuadorian CBS. Variance explained by first and second components of PCA is shown in parenthesis for PC1 and PC2 in each graph. Boxplots showing abundance of two key polyphenolic compounds (PCB and PCB trimer 6) (**b**) and TPC, TTC, and TFC (**d**) that can be used as possible markers for separation based on CBS variety are shown next to their correspondent PCA graph. For interpretation of legends, see Table 1.

In this section, CBS data from one representative country of cocoa production (Ecuador), with one of the most representative numbers of samples among our sample set (*n* = 7) and offering CBS samples belonging to the 4 varieties, were used. The aim was to verify whether both HPLC and spectrophotometric assays were able to classify CBS samples among one same country depending on the cocoa variety. Although it was previously observed that variety differentiation was not possible when taking into account all the samples from our sample set or all the samples from one single continent, it was found that this differentiation was possible if samples from just one country were analyzed. The separation of CBS samples from Ecuador according to their varieties showed a better discrimination of the samples by both HPLC and spectrophotometric results (Figure 4a,c, respectively). 

18 out of the 25 determined compounds showed significant differences among the four different CBS varieties (Table S5). Boxplots corresponding to PCB and PCB trimer 6 were chosen for representation for being the marker compounds that gave the highest number of significant differences among comparisons and for being compounds with high concentrations in the analyzed CBS (Figure 4b). On the one hand, PCB was found to be in high concentrations in the Ecuadorian Forastero CBSs, which allowed to differentiate this variety from the Criollo CBS that presented the lowest concentration of this compound. On the other hand, PCB trimer 6 allowed to separate the four varieties, being more present in the Nacional variety, followed by the Forastero, Criollo, and Trinitario CBS. Regarding the separation by spectrophotometric analysis results, the three studied parameters gave proportional results for the four CBS varieties (Figure 4d). These techniques allowed to separate the Forastero, Trinitario, and Criollo varieties, while the Nacional variety clustered together with Forastero and Criollo in TPC, with Forastero in TTC, and with both Forastero and Criollo in TFC. The Nacional variety belongs to the Forastero variety from a botanical point of view [21], which would explain the clusterization of these boxplots. Forastero CBSs seemed to be the ones with the highest polyphenol concentrations among the four Ecuadorian varieties, which confirms in this case the observations previously described for the variety of cocoa beans [3].

### 3.4. Classification of CBS Based on Their Methylxanthine Ratio According to Variety

**Figure 5:**
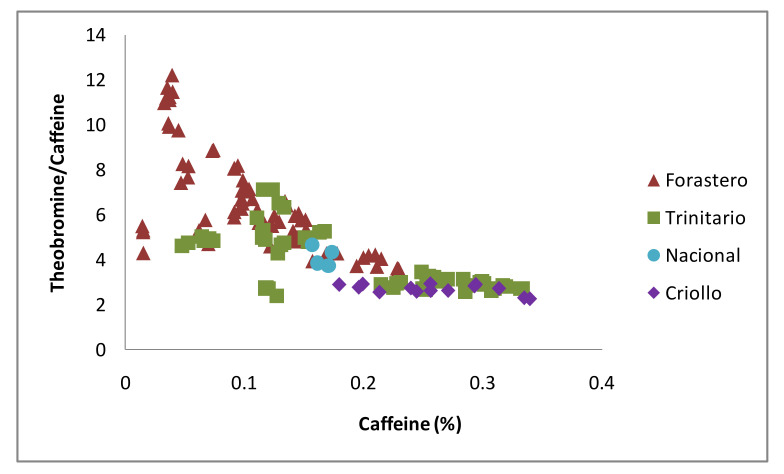
Relationship between methylxanthine content in CBS and CBS variety.

Methylxanthine content in cocoa beans, and concretely, the ratio between theobromine and caffeine contents, were previously reported to be dependent on the cocoa variety. Studies carried out by Davrieux et al. [45] reported a classification of cocoa samples into Forastero, Trinitario, and Criollo varieties depending on the methylxanthine ratio determined by near infrared spectroscopy. After that, other works concerning the study of cocoa bean methylxanthine content through HPLC [17,33] and HPTLC [27] reported similar classifications of cocoa varieties when building a graph in which theobromine/caffeine concentration ratio is represented in axe Y and the percentage of caffeine content in the sample is represented in axe X. A similar graph was built in the present study with the results of the methylxanthine content obtained for the different CBS samples and is shown in Figure 5. 

For the first time for the cocoa byproduct, a variety distribution depending on the methylxanthine content is reported. This distribution is similar to the one observed before for cocoa beans and allow to classify the different CBS varieties. Forastero samples seemed to have high concentrations of theobromine and low concentrations of caffeine, which situated these samples in the upper-left corner of the graph. On the other hand, Criollo CBS presented the opposite characteristics; they had lower content of theobromine and higher content of caffeine, appearing in the lower-right corner of the graph. Trinitario variety is a hybrid from the two previously mentioned varieties [21], and samples belonging to this group appeared in an intermediate position of the graph. Finally, as it was previously mentioned, Nacional variety belongs to the Forastero group from a botanical point of view [21], and CBS samples belonging to Nacional variety were therefore found together with the Forastero group. The average theobromine/caffeine ratios for the CBS samples were 6.45, 4.15, 3.88, and 2.69 for Forastero, Nacional, Trinitario, and Criollo varieties, respectively. These values are in accordance to those obtained by Davrieux et al. [45] for cocoa beans (7.3 for Forastero, 4.0 and 3.6 for Trinitario, and 1.5 for Criollo variety). While the HPLC-determined polyphenolic content did not allow for a separation of the complete sample set of CBSs depending on their varieties, this finding could allow for a rapid variety classification of CBS depending on their HPLC-determined theobromine and caffeine content.

## 4. Conclusions

This study provides information about the polyphenolic profile of CBS, obtained by both RP-HPLC-PDA Analysis and Spectrophotometric Assays for a large set of samples. Different cocoa polyphenolic markers were found such as catechin, epicatechin, procyanidins, quercetin glycosides, and *N*-phenylpropenoyl-L-aminoacids, among others. CBS was therefore demonstrated to have a similar polyphenolic profile to that of cocoa beans. Generally, CBS polyphenolic concentrations were lower than those found for cocoa beans. Some compounds such as monomeric flavanols and *N*-phenylpropenoyl-L-aminoacids were found in CBS in concentrations that were an order of magnitude below those of cocoa powder. However, other polyphenolic compounds such as protocatechuic acid were found in higher concentrations in CBS than in that of cocoa powder. Individual polyphenolic compounds concentration showed high variability among the CBS samples set in the present study, which was related to the different geographic origin of samples (different cultivation traditions and climates) rather than their different varieties.

Spectrophotometric analyses of the CBS polyphenolic content demonstrated TPC, TFC, TFC, and RSA to be powerful tools for polyphenol content comparison among samples. The spectrophotometric results were shown to be highly correlated with the HPLC-determined results. However, these parameters highly overestimated polyphenol content in CBS and should therefore be used with precaution, and preferably always supported by chromatographic techniques that allow to establish structure-activity relationships. In this frame, it was also found that the obtained values of RSA (antioxidant capacity) were highly correlated with the HPLC-determined total polyphenolic content and, concretely, PCA glycosides and PCB trimers seemed to be the compound groups contributing the most to this parameter.

Chemometric analysis of the CBS polyphenolic contents through PCA did not allow for CBS sample differentiation according to variety when taking into account our whole CBS sample set. Nevertheless, it was possible to differentiate CBS samples according to their continent of origin (America or Africa). This was possible thanks to some marker compounds such as catechin and PCB trimer 6 that were significantly more present in American samples than in African samples. The same separation was also obtained with the spectrophotometric results, with American CBSs presenting higher values of TPC, TFC, and TTC than those of African samples. This finding has a great utility for CBS origin traceability from an instrumental point of view since similar differentiation results can be obtained by using screening, rapid, and easy to handle methods such the spectrophotometric assays instead of more sophisticated methods such as chromatography techniques. On the other hand, marker compounds such as epicatechin and PCB trimer 4 allowed for a differentiation among African CBS according to the country of origin, whereas protocatechuic acid and *N*-caffeoyl-L-aspartate served for the classification of American CBS according to the country of origin. In these latest cases, similar differentiation results could again be obtained by using spectrophotometric techniques.

Finally, it was found that the classification of the whole CBS sample set according to their variety, which could not be obtained with the polyphenolic profile, could be achieved by analyzing their theobromine/caffeine ratio. This ratio was shown to be the highest for Forastero CBS, followed Nacional, Trinitario, and Criollo, which was the variety with the lowest theobromine/caffeine ratio. This finding was previously reported for cocoa beans and, for the first time, the equivalent was successfully applied for CBS in the present study.

CBS was recently proposed as a high-added value ingredient for functional foods and, therefore, its polyphenolic and methylxanthine contents are important parameters to this end. The present study demonstrates that CBS is an interesting ingredient to yield polyphenolic and methylxanthine rich products by being recycled inside the cocoa manufacturing industry in the frame of a circular economy and offers interesting tools for the selection of the byproduct in this context.

## Figures and Tables

**Table 1:** Origin of fermented and dried cocoa beans used to yield cocoa bean shells.

Sample Code	Variety	Country	Continent
BRA	Trinitario	Brazil	America
CAM1	Forastero	Cameroon	Africa
CAM2	Trinitario	Cameroon	Africa
COL1	Forastero	Colombia	America
COL2	Trinitario	Colombia	America
CON1	Forastero	Congo	Africa
CON2	Forastero	Congo	Africa
DOR1	Trinitario	Dominican Republic	America
DOR2	Forastero	Dominican Republic	America
DOR3	Trinitario	Dominican Republic	America
DOR4	Trinitario	Dominican Republic	America
ECU1	Forastero	Ecuador	America
ECU2	Trinitario	Ecuador	America
ECU3	Forastero	Ecuador	America
ECU4	Nacional	Ecuador	America
ECU5	Nacional	Ecuador	America
ECU6	Forastero	Ecuador	America
ECU7	Criollo	Ecuador	America
GHA	Forastero	Ghana	Africa
IVC	Forastero	Ivory Coast	Africa
JAM	Trinitario	Jamaica	America
MAD	Forastero	Madagascar	Africa
MEX	Trinitario	Mexico	America
PER1	Forastero	Peru	America
PER2	Trinitario	Peru	America
SAT1	Forastero	São Tomé	Africa
SAT2	Forastero	São Tomé	Africa
SAT3	Forastero	São Tomé	Africa
SLE	Forastero	Sierra Leone	Africa
TAN	Forastero	Tanzania	Africa
TOG1	Forastero	Togo	Africa
TOG2	Forastero	Togo	Africa
UGA1	Forastero	Uganda	Africa
UGA2	Forastero	Uganda	Africa
VEN1	Trinitario	Venezuela	America
VEN2	Trinitario	Venezuela	America
VEN3	Trinitario	Venezuela	America
VEN4	Trinitario	Venezuela	America
VEN5	Criollo	Venezuela	America
VEN6	Trinitario	Venezuela	America
VEN7	Criollo	Venezuela	America
VEN8	Criollo	Venezuela	America
VEN9	Criollo	Venezuela	America
VEN10	Criollo	Venezuela	America

**Table 2:** Identification and concentration range of polyphenolic compounds and methylxanthines in CBS extracts yielded from roasted cocoa beans from different origins and cultivars.

Peak	R_t_ (min)	λ_max_	Compound	Concentration Range (mg kg^−1^ of CBS)
1	4.73	293	Protocatechuic acid	11.89‒241.01
2	6.4	272	Theobromine	764.88‒9028.28
3	6.8	279	*N*-Coumaroyl-L-aspartate_1	1.41‒24.55
4	8.24	320	*N*-Caffeoyl-L-aspartate	5.86‒652.58
5	8.63	278	Catechin	12.72‒180.37
6	9.5	279	Catechin-3-O-glucoside	6.40‒101.49
7	9.7	280	Procyanidin B-type trimer_1	0.90‒130.89
8	10.6	306	*N*-Coumaroyl-L-aspartate_2	4.46‒184.30
9	11.08	278	Procyanidin B-type trimer_2	0.00‒163.15
10	11.1	285	Procyanidin B-type	6.84‒672.55
11	11.5	278	Epicatechin	4.47‒748.79
12	11.87	286	*N*-Coumaroyl-L-glutamate	1.10‒12.58
13	12.2	272	Caffeine	150.81‒3370.77
14	12.78	279	Procyanidin B-type trimer_3	1.51‒97.04
15	13	278	Procyanidin B-type trimer_4	7.62‒264.48
16	13.22	281	Procyanidin B-type trimer_5	6.52‒280.29
17	13.86	280	Procyanidin A-type hexoside_1	8.51‒560.65
18	14.6	279	Procyanidin B-type trimer_6	0.00‒28.68
19	14.9	280	Procyanidin A-type pentoside_1	1.27‒198.93
20	15.82	279	Procyanidin B-type trimer_7	2.49‒104.37
21	16.03	279	Procyanidin A-type trimer arabinoside	2.44‒114.09
22	17.2	278	Procyanidin A-type hexoside_2	2.49‒73.34
23	17.5	283	*N*-Coumaroyl-L-tyrosine	1.99‒48.70
24	17.7	278	Procyanidin A-type pentoside_2	0.00‒40.02
25	18.2	355	Quercetin-3-O-glucoside	3.47‒47.59
26	19.4	355	Quercetin-3-O-arabinoside	3.76‒86.53
27	21.83	365	Quercetin	1.07‒22.37

**Table 3:** Sums (∑) for total contents of different polyphenolic categories, methylxanthines, and spectrophotometric assays values determined in CBS powders from different geographical origins and varieties. Concentrations are expressed as mg/kg CBS, unless indicated differently. An extended version of this table with the individual compound concentrations can be consulted in Table S1 of the Supplementary Materials. Data are presented as mean (*n* = 4) ± standard deviation.

Sample Code	∑ Phenolic Acids	∑ Flavan-3- ols	∑ Catechin- 3-O- glycosides	∑ PCB	∑ PCB Trimers	∑ PCA Glycosides	∑ Flavonols	∑ Flavonol-3- O-glycosides	∑ *N*-phenyl -propenoyl-L- aminoacids	∑ Total Polyphenols	∑ Total Methylxantines (g/kg CBS)	TPC (mg GAE/g CBS)	TFC (mg CE/g CBS)	TTC (mg CE/g CBS)	RSA (μmol TE/g CBS)
BRA	11.9 ± 0.8	58 ± 5	31 ± 3	9.8 ± 0.8	195 ± 10	103 ± 4	305 ± 03	39 ± 3	41 ± 1	493 ± 13	4.1 ± 0.2	18.1 ± 0.9	4.9 ± 0.2	2.8 ± 0.2	60 ± 3
CAM1	112 ± 3	116 ± 2	32 ± 1	68 ± 8	181 ± 4	225 ± 5	3.4 ± 0.2	30.6 ± 0.1	105 ± 2	874 ± 11	7.3 ± 0.1	18 ± 1	6.1 ± 0.3	4.8 ± 0.2	78 ± 5
CAM2	28 ± 2	57 ± 5	16.8 ± 0.7	16.1 ± 0.4	46 ± 2	32 ± 2	1.1 ± 0.1	12.2 ± 0.5	29 ± 1	238 ± 6	3.2 ± 0.3	12.2 ± 0.8	5.0 ± 0.1	3.4 ± 0.1	54 ± 1
COL1	122 ± 5	344 ± 22	26 ± 1	210 ± 21	363 ± 18	339 ± 6	6.6 ± 0.5	56 ± 4	220 ± 8	1686 ± 37	7.3 ± 0.4	28.0 ± 0.6	12.4 ± 0.3	7.7 ± 0.6	119 ± 6
COL2	130 ± 8	859 ± 12	41 ± 4	289 ± 29	650 ± 34	497 ± 26	9 ± 1	89 ± 5	543 ± 12	3108 ± 56	10.8 ± 0.1	35 ± 1	16.7 ± 0.9	8.1 ± 0.4	144 ± 9
CON1	138 ± 8	86.0 ± 0.6	16.2 ± 0.6	274 ± 30	150 ± 3	128 ± 4	3.3 ± 0.4	47 ± 2	197 ± 10	1040 ± 33	8.4 ± 0.4	16.1 ± 0.1	5.81 ± 0.03	3.0 ± 0.1	64.24 ± 0.02
CON2	141 ± 9	180 ± 9	67 ± 4	141 ± 16	238 ± 9	271 ± 14	5.5 ± 0.3	41 ± 4	161 ± 9	1244 ± 29	8.4 ± 0.2	17.9 ± 0.5	7.3 ± 0.3	4.1 ± 0.1	76 ± 2
DOR1	176 ± 9	588 ± 45	71 ± 4	584 ± 21	601 ± 15	425 ± 17	10.3 ± 0.7	94 ± 4	584 ± 15	3134 ± 57	12.3 ± 0.1	34.0 ± 0.5	17.7 ± 0.2	8.0 ± 0.3	146 ± 3
DOR2	136 ± 10	245 ± 11	39 ± 3	275 ± 21	305 ± 9	296 ± 15	3.1 ± 0.2	72 ± 4	459 ± 27	1831 ± 41	9.3 ± 0.4	21 ± 2	6.9 ± 0.6	5.0 ± 0.2	79 ± 4
DOR3	131 ± 3	227 ± 23	13.8 ± 0.9	78 ± 11	252 ± 10	184 ± 9	1.4 ± 0.1	19.3 ± 0.7	68 ± 2	975 ± 29	2.9 ± 0.2	17.8 ± 0.5	5.6 ± 0.3	7.1 ± 0.2	78.46 ± 0.03
DOR4	141 ± 6	288 ± 18	48 ± 2	236 ± 26	276 ±10	310 ± 11	7.0 ± 0.3	62 ± 3	395 ± 24	1762 ± 43	11.1 ± 0.2	18 ± 1	6.6 ± 0.3	3.5 ± 0.2	74 ± 5
ECU1	99 ± 9	250 ± 20	25 ± 2	310 ± 23	216 ± 9	302 ± 31	2.6 ± 0.1	35 ± 2	155 ± 8	1394 ± 46	6.8 ± 0.5	19 ± 2	6.8 ± 0.2	4.8 ± 0.4	71 ± 4
ECU2	122 ± 9	214 ± 8	12.2 ± 0.7	167 ± 14	162 ± 9	122 ± 4	3.1 ± 0.3	23.8 ± 0.6	93 ± 4	919 ± 21	7.2 ± 0.3	12 ± 1	4.1 ± 0.2	3.0 ± 0.1	53 ± 2
ECU3	206 ± 19	480 ± 13	48 ± 1	233 ± 12	476 ± 13	496 ± 22	9.9 ± 0.9	112 ± 2	667 ± 34	2728 ± 50	10.7 ± 0.3	35.8 ± 0.8	19 ± 1	7.9 ± 0.5	150 ± 6
ECU4	145 ± 2	414 ± 14	22 ± 1	198 ± 10	248 ± 5	306 ± 12	6.9 ± 0.4	56.6 ± 0.7	213 ± 7	1610 ± 23	9.1 ± 0.2	20 ± 2	7.2 ± 0.6	5.8 ± 0.5	84 ± 8
ECU5	113 ± 8	287 ± 1	27.2 ± 0.1	96 ± 8	214 ± 14	294 ± 8	3.0 ± 0.3	37 ± 2	160 ± 2	1231 ± 20	8.0 ± 0.1	14.7 ± 0.2	4.7 ± 0.2	4.1 ± 0.1	59.9 ± 0.3
ECU6	88 ± 5	462 ± 8	15.6 ± 0.2	277 ± 28	298 ± 14	371 ± 18	6.1 ± 0.6	36 ± 2	264 ± 10	1818 ± 39	8.1 ± 0.3	18.8 ± 0.8	8.1 ± 0.3	4.5 ± 0.3	78 ± 4
ECU7	108 ± 3	437 ± 13	37 ± 3	52 ± 2	284 ± 7	292 ± 12	2.8 ± 0.2	65.4 ± 0.8	277 ± 6	1556 ± 20	10.8 ± 0.8	14.6 ± 0.1	6.4 ± 0.3	3.8 ± 0.2	62.4 ± 0.9
GHA	20.9 ± 0.8	17.2 ± 0.6	6.8 ± 0.1	6.8 ± 0.4	26 ± 1	16.3 ± 0.6	1.29 ± 0.03	7.2 ± 0.4	17.1 ± 0.7	120 ± 2	0.9 ± 0.1	9.1 ± 0.6	3.1 ± 0.1	2.3 ± 0.1	39 ± 1
IVC	54 ± 4	77 ± 7	21 ± 1	46 ± 5	74 ± 3	68 ± 2	1.1 ± 0.1	13.4 ± 0.8	50 ± 2	405 ± 10	4.2 ± 0.3	10.0 ± 0.8	3.2 ± 0.3	2.5 ± 0.2	44 ± 4
JAM	187 ± 18	245 ± 16	58 ± 2	57 ± 6	455 ± 17	287 ± 16	6.9 ± 0.9	76 ± 1	450 ± 17	1822 ± 39	11.9 ± 0.2	20 ± 2	8.6 ± 0.3	3.7 ± 0.2	82 ± 7
MAD	87 ± 4	888 ± 63	31.5 ± 0.6	478 ± 19	518 ± 20	412 ± 9	6.9 ± 0.1	50 ± 2	371 ± 4	2843 ± 70	10.55 ± 0.02	3.0 ± 0.5	12.5 ± 0.4	7.7 ± 0.2	139 ± 1
MEX	241 ± 6	523 ± 13	89 ± 4	261 ± 35	538 ± 23	658 ± 38	22.4 ± 0.6	93 ± 4	810 ± 40	3235 ± 71	9.8 ± 0.3	39 ± 2	18 ± 2	9.3 ± 0.1	172 ± 5
PER1	120 ± 7	413 ± 30	27 ± 2	138 ± 2	272 ± 10	417 ± 20	7.5 ± 0.3	55 ± 1	311 ± 16	1759 ± 41	8.3 ± 0.	26 ± 2	13.4 ± 0.6	6.7 ± 0.6	116 ± 8
PER2	87 ± 3	385 ± 42	33 ± 2	341 ± 26	340 ± 14	351 ± 8	6.3 ± 0.5	51.9 ± 0.7	404 ± 10	1999 ± 53	9.7 ± 0.7	43 ± 2	18 ± 1	11.0 ± 0.5	182 ± 6
SAT1	120 ± 4	352 ± 29	42 ± 1	640 ± 41	484 ± 20	387 ± 21	4.7 ± 0.3	38 ± 2	203 ± 13	2271 ± 59	8.9 ± 0.3	26 ± 2	10.9 ± 0.8	5.3 ± 0.4	103 ± 9
SAT2	198 ± 11	751 ± 20	101 ± 3	191 ± 20	654 ± 36	833 ± 21	13.9 ± 0.5	122 ± 6	539 ± 31	3403 ± 61	10.1 ± 0.2	34 ± 1	16.7 ± 0.4	10.9 ± 0.3	155 ± 8
SAT3	164 ± 16	656 ± 46	79 ± 3	246 ± 30	566 ± 32	682 ± 61	8.0 ± 0.9	62 ± 2	406 ± 11	2869 ± 90	10.0 ± 0.3	38 ± 1	20 ± 1	10.4 ± 0.1	165 ± 6
SLE	14.4 ± 0.7	225 ± 14	29 ± 2	136 ± 12	199 ± 11	129 ± 10	2.0 ± 0.1	28 ± 1	61 ± 1	823 ± 24	4.7 ± 0.3	21.0 ± 0.6	6.7 ± 0.1	5.5 ± 0.1	73 ± 1
TAN	88 ± 7	173 ± 18	17.9 ± 0.7	253 ± 18	263 ± 17	199 ± 16	2.9 ± 0.2	32 ± 2	93 ± 4	1123 ± 35	8.0 ± 0.5	13.1 ± 0.9	3.1 ± 0.2	3.6 ± 0.3	55 ± 4
TOG1	14 ± 1	43 ± 3	27 ± 2	136 ± 12	154 ± 7	73 ± 3	1.1 ± 0.1	24 ± 1	61 ± 2	534 ± 15	4.8 ± 0.3	12.7 ± 0.9	3.0 ± 0.1	2.0 ± 0.1	45 ± 2
TOG2	16 ± 1	58 ± 2	28 ± 2	146 ± 14	123 ± 5	85 ± 3	1.4 ± 0.1	24 ± 2	45 ± 2	526 ± 16	4.1 ± 0.1	14 ± 1	3.0 ± 0.3	3.0 ± 0.2	49 ± 3
UGA1	57 ± 3	45 ± 3	6.4 ± 0.3	129 ± 5	76 ± 5	45 ± 3	2.0 ± 0.1	16.3 ± 0.7	61 ± 2	438 ± 9	4.5 ± 0.4	9.0 ± 0.6	2.5 ± 0.2	2.1 ± 0.2	37 ± 3
UGA2	108 ± 8	67 ± 5	13 ± 1	38 ± 5	97 ± 4	48 ± 4	2.4 ± 0.2	15.8 ± 0.5	58 ± 2	449 ± 12	7.8 ± 0.3	6.1 ± 0.2	1.4 ± 0.1	1.1 ± 0.1	28 ± 2
VEN1	119 ± 5	314 ± 26	16.8 ± 0.7	408 ± 53	330 ± 14	259 ± 10	3.2 ± 0.3	28 ± 1	104 ± 4	1583 ± 62	7.3 ± 0.4	22 ± 1	7.7 ± 0.8	6.9 ± 0.2	98 ± 6
VEN2	73 ± 2	195 ± 19	14 ± 1	673 ± 52	617 ± 36	179 ± 6	5.9 ± 0.8	42 ± 2	217 ± 16	1136 ± 68	8.8 ± 0.4	27.5 ± 0.1	8.7 ± 0.3	4.9 ± 0.4	99 ± 1
VEN3	108 ± 4	412 ± 31	52 ± 5	78 ± 4	377 ± 12	467 ± 16	3.4 ± 0.3	76 ± 4	319 ± 11	2015 ± 40	11.2 ± 0.6	20 ± 2	8.9 ± 0.6	5.1 ± 0.3	87 ± 4
VEN4	46 ± 3	171 ± 5	20 ± 1	83 ± 3	126 ± 3	98 ± 4	2.6 ± 0.1	21.5 ± 0.7	133 ± 9	1892 ± 13	4.4 ± 0.0	20 ± 1	8.9 ± 0.7	4.5 ± 0.1	82 ± 4
VEN5	121 ± 9	164 ± 12	25 ± 1	55 ± 3	220 ± 7	127 ± 3	3.3 ± 0.3	33.0 ± 0.9	188 ± 6	702 ± 18	8.3 ± 0.5	15 ± 1	5.9 ± 0.3	3.5 ± 0.3	66 ± 4
VEN6	124 ± 9	256 ± 16	30 ± 2	182 ± 10	375 ± 17	252 ± 16	6.1 ± 0.5	49 ± 2	281 ± 17	937 ± 36	9.9 ± 0.7	22 ± 2	9.1 ± 0.2	4.5 ± 0.2	86 ± 8
VEN7	104 ± 4	319 ± 5	34.8 ± 0.5	106 ± 9	342 ± 8	267 ± 3	1.66 ± 0.02	27 ± 1	322 ± 5	1555 ± 15	11.11 ± 0.05	14.9 ± 0.7	5.6 ± 0.4	2.4 ± 0.2	55.6 ± 0.5
VEN8	136 ± 4	347 ± 7	50 ± 3	54 ± 2	286 ± 10	329 ± 21	2.94 ± 0.04	58 ± 1	322 ± 5	1523 ± 25	11.4 ± 0.2	18.5 ± 0.7	6.2 ± 0.5	3.3 ± 0.1	68 ± 2
VEN9	86 ± 5	215 ± 7	25 ± 2	68 ± 5	208 ± 8	230 ± 14	3.5 ± 0.4	24 ± 1	178 ± 4	1584 ± 20	9.6 ± 0.3	12.5 ± 0.1	2.8 ± 0.1	2.17 ± 0.05	47 ± 3
VEN10	12.05 ± 0.02	306 ± 2	32.9 ± 0.6	104 ± 12	220 ± 4	209 ± 11	2.4 ± 0.1	36 ± 1	214 ± 7	1036 ± 18	7.2 ± 0.2	19.4 ± 0.9	5.0 ± 0.4	5.4 ± 0.1	65 ± 1

PCB = procyanidin B-type, PCA = procyanidin A-Type, TPC = total phenolic content, GAE = gallic acid equivalents, TFC = total flavonoid content, CE = catechin equivalents, TTC = total tannin content, RSA = radical scavenging activity, TE = trolox equivalents.

## Data Availability

The data supporting the findings of this study are available in this Article and its Supplementary Materials File.

## Supplementary Materials

The following are available online at https://www.mdpi.com/article/10.3390/antiox10101533/s1, Figure S1: RP-HPLC-PDA chromatograms of a representative CBS extract recorded at 272 nm, 278 nm, and 356 nm; Table S1: Content of the single identified polyphenolic compounds and methylxantines in CBS powders from different geographical origins and cultivars; Table S2: Pairwise comparison of the phenolic compounds quantified in CBSs by RP-HPLC-PDA that could be used as potential geographical origin markers among the American and African continents; Table S3: Pairwise comparison of the phenolic compounds quantified in CBSs by RP-HPLC-PDA that could be used as potential geographical origin markers for different countries within the African continent; Table S4: Pairwise comparison of the phenolic compounds quantified in CBSs by RP-HPLC-PDA that could be used as potential geographical origin markers for different countries within the American continent; Table S5: Pairwise comparison of the phenolic compounds quantified in CBSs by RP-HPLC-PDA that could be used as potential markers for cultivars among Ecuadorian samples.
