# Supplementary material for: Chemometric Classification of Cocoa Bean Shells Based on Their Polyphenolic Profile Determined by RP-HPLC-PDA Analysis and Spectrophotometric Assays
Source: Antioxidants (Basel). 2021 Sep 27;10(10):1533. doi: 10.3390/antiox10101533 (PMC8532815; doi:10.3390/antiox10101533)
Supplement: Supplementary file 1 [file antioxidants-10-01533-s001.zip › antioxidants-1350936-supplementary.pdf]

antioxidants

MDPI

Article Supplementary Material

Chemometric Classification of Cocoa Bean Shells Based on their Polyphenolic Profile Determined by RP-HPLC-PDA Analysis and Spectrophotometric Assays

Olga Rojo-Poveda [1,2,*], Giuseppe Zeppa [1], Ilario Ferrocino [1], Caroline Stévigny [2], and Letricia Barbosa-Pereira [1,3]

1 Department of Agriculture, Forestry and Food Sciences (DISAFA), University of Turin, 10095 Grugliasco, Italy; giuseppe.zeppa@unito.it (G.Z.); ilario.ferrocino@unito.it (I.F.); letricia.barbosa.pereira@usc.es (L.B.-P.)
2 RD3 Department-Unit of Pharmacognosy, Bioanalysis and Drug Discovery, Faculty of Pharmacy, Université libre de Bruxelles, 1050 Brussels, Belgium; Caroline.Stevigny@ulb.be
3 Department of Analytical Chemistry, Nutrition and Food Science, Faculty of Pharmacy, University of Santiago de Compostela, 15782 Santiago de Compostela, Spain
* Correspondence: Olga.Rojo.Poveda@ulb.be

Abstract: The cocoa bean shell (CBS), a by-product from the cocoa industry, has been recently proposed as a functional and low-cost ingredient, mainly because of its content in polyphenols. However, vegetal food products could significantly differ in their chemical composition depending on different factors such as their geographical provenience. This work is aimed to determine the polyphenolic and methylxanthine profile of different CBS samples and utilize it for achieving their differentiation according to their geographical origin and variety. RP-HPLC-PDA was used to determine the CBS polyphenolic profile. Spectrophotometric assays were used to obtain the total phenolic, flavonoid, and tannin contents, as well as to evaluate their radical scavenging activity. The results obtained from both methods were then compared and used for the CBS differentiation according to their origin and varieties through chemometric analysis. RP-HPLC-PDA allowed to determine 25 polyphenolic compounds, as well as the methylxanthines theobromine and caffeine. Polyphenolic profile results highlighted significant differences among the analyzed samples, allowing for their differentiation based on their geographical provenience. Similar results were achieved with the results of the spectrophotometric assays, considered as screening methods. Differentiation based on CBS variety was instead obtained based on the HPLC-determined methylxanthine profile.

Keywords: cocoa bean shell; cocoa by-product; RP-HPLC-PDA; spectrophotometric assays; radical scavenging activity; principal component analysis; polyphenols; methylxanthines, fingerprints; cocoa markers

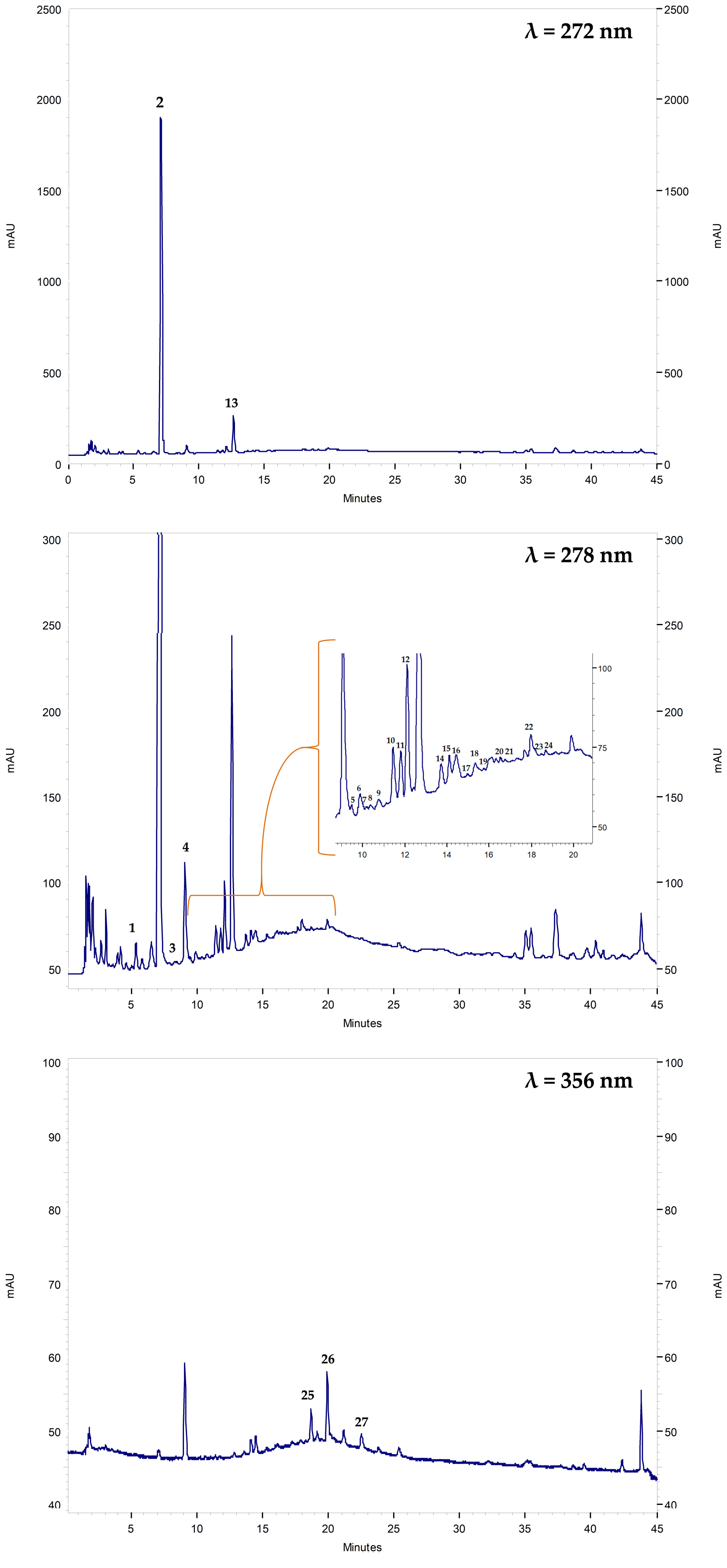

Figure S1. RP-HPLC-PDA chromatograms of a representative CBS extract recorded at 272 nm, 278 nm, and 356 nm

Table S1. Content of the single identified polyphenolic compounds and methylxantines in CBS powders from different geographical origins and cultivars. Data are presented as the mean (n=4) ± standard deviation.

**POLYPHENOLS**	**Concentration (mg kg-1 of CBS)**										
	BRA	CAM1	CAM2	COL1	COL2	CON1	CON2	DOR1	DOR2	DOR3	DOR4
**Phenolic acids**											
Protocatechuic acid	11.9 ± 0.8	112 ± 3	28 ± 2	122 ± 5	130 ± 8	138 ± 8	141 ± 9	176 ± 9	136 ± 10	131 ± 3	141 ± 6
Σ	**11.9 ± 0.8**	**112 ± 3**	**28 ± 2**	**122 ± 5**	**130 ± 8**	**138 ± 8**	**141 ± 9**	**176 ± 9**	**136 ± 10**	**131 ± 3**	**141 ± 6**
**Flavan-3-ol**											
Catechin	15 ± 1	20 ± 2	13.5 ± 0.5	66 ± 6	128 ± 7	14.2 ± 0.1	26 ± 2	95 ± 2	76 ± 6	46 ± 2	59 ± 5
Epicatechin	44 ± 4	96 ± 1	43 ± 5	279 ± 21	731 ± 10	72 ± 1	154 ± 9	493 ± 45	169 ± 9	181 ± 23	229 ± 17
Σ	**58 ± 5**	**116 ± 2**	**57 ± 5**	**344 ± 22**	**859 ± 12**	**86 ± 1**	**180 ± 9**	**588 ± 45**	**245 ± 11**	**227 ± 23**	**288 ± 18**
**Catechin-3-O-glycosides**											
Catechin-3-O-glucoside	31 ± 3	32 ± 1	17 ± 1	26 ± 1	41 ± 4	16.2 ± 0.6	67 ± 4	71 ± 4	39 ± 3	13.7 ± 0.9	48 ± 2
Σ	**31 ± 3**	**32 ± 1**	**17 ± 1**	**26 ± 1**	**41 ± 4**	**16.2 ± 0.6**	**67 ± 4**	**71 ± 4**	**39 ± 3**	**13.7 ± 0.9**	**48 ± 2**
**Procyanidins B-type (PCB)**											
PCB	9.8 ± 0.8	68 ± 8	16.1 ± 0.4	210 ± 21	289 ± 29	274 ± 30	141 ± 16	584 ± 21	275 ± 21	78 ± 11	236 ± 26
Σ	**9.8 ± 0.8**	**68 ± 8**	**16.1 ± 0.4**	**210 ± 21**	**289 ± 29**	**274 ± 30**	**141 ± 16**	**584 ± 21**	**275 ± 21**	**78 ± 11**	**236 ± 26**
**Procyanidins B-type (PCB) trimers**											
PCB trimer 1	48 ± 4	11.2 ± 0.1	0.9 ± 0.1	34 ± 2	74 ± 5	19.9 ± 0.2	13.3 ± 0.6	76 ± 3	54 ± 4	12.8 ± 0.9	48 ± 6
PCB trimer 2	4.8 ± 0.3	3.99 ± 0.03	3.1 ± 0.2	3.0 ± 0.2	23 ± 1	4.6 ± 0.1	8.9 ± 0.7	37 ± 3	34 ± 3	2.0 ± 0.2	19 ± 2
PCB trimer 3	10 ± 1	14 ± 2	1.6 ± 0.2	8.3 ± 0.5	3.4 ± 0.3	18.5 ± 0.4	20 ± 2	14 ± 1	17 ± 2	20.7 ± 0.5	12 ± 2
PCB trimer 4	32 ± 3	90 ± 2	22.7 ± 0.7	140 ± 14	200 ± 21	38 ± 2	81 ± 7	126 ± 12	65 ± 4	114 ± 9	85 ± 4
PCB trimer 5	79 ± 9	32 ± 3	14 ± 2	128 ± 11	280 ± 26	44 ± 2	82 ± 6	235 ± 3	87 ± 6	53 ± 3	57 ± 6
PCB trimer 6	1.7 ± 0.1	8.0 ± 0.9	1.1 ± 0.2	10 ± 1	12 ± 1	1.45 ± 0.03	6.7 ± 0.6	18 ± 2	7.1 ± 0.6	28.7 ± 0.9	12.6 ± 0.8
PCB trimer 7	21 ± 2	23 ± 1	2.5 ± 0.1	40 ± 5	58 ± 5	22.8 ± 0.9	25 ± 1	95 ± 7	41 ± 3	21.0 ± 0.2	42 ± 3
Σ	**195 ± 10**	**181 ± 4**	**46 ± 2**	**363 ± 18**	**650 ± 34**	**150 ± 3**	**238 ± 9**	**601 ± 15**	**305 ± 9**	**252 ± 10**	**276 ± 10**
**Procyanidins A-type (PCA) glycosides**											
PCA hexoside 1	41 ± 3	143 ± 4	16 ± 1	218 ± 3	284 ± 24	70 ± 3	165 ± 13	178 ± 14	155 ± 14	141 ± 9	198 ± 10
PCA pentoside 1	20 ± 2	17.9 ± 0.8	3.2 ± 0.4	30 ± 1	65 ± 8	21.3 ± 0.9	15.1 ± 0.4	66 ± 4	61 ± 6	19 ± 2	36 ± 3
PCA trimer arabinoside	13 ± 2	20.0 ± 0.9	2.4 ± 0.3	34 ± 2	69 ± 5	18 ± 2	35 ± 2	83 ± 9	36 ± 4	14 ± 2	34 ± 1
PCA hexoside 2	20 ± 1	30.0 ± 0.8	6.9 ± 0.4	33 ± 3	54 ± 2	14 ± 1	37 ± 3	69 ± 3	34 ± 2	8.2 ± 0.4	28 ± 3
PCA pentoside 2	9.5 ± 0.3	14.6 ± 0.5	3.56 ± 0.02	23 ± 3	25 ± 3	4.8 ± 0.4	18 ± 2	28 ± 1	9.7 ± 0.7	1.7 ± 0.1	13.5 ± 0.8
Σ	**103 ± 4**	**225 ± 5**	**32 ± 2**	**339 ± 6**	**497 ± 26**	**128 ± 4**	**271 ± 14**	**425 ± 17**	**296 ± 15**	**184 ± 9**	**310 ± 11**
**Flavonols**											
Quercetin	3.5 ± 0.3	3.4 ± 0.2	1.1 ± 0.1	6.6 ± 0.5	9 ± 1	3.3 ± 0.4	5.5 ± 0.3	10.3 ± 0.7	3.1 ± 0.2	1.4 ± 0.1	7.0 ± 0.3
Σ	**3.5 ± 0.3**	**3.4 ± 0.2**	**1.1 ± 0.1**	**6.6 ± 0.5**	**9 ± 1**	**3.3 ± 0.4**	**5.5 ± 0.3**	**10.3 ± 0.7**	**3.1 ± 0.2**	**1.4 ± 0.1**	**7.0 ± 0.3**
**Flavonol-3-O-glycosides**											
Quercetin-3-O-glucoside	6.4 ± 0.4	8.41 ± 0.05	5.1 ± 0.3	23 ± 2	37 ± 3	17.7 ± 0.5	12.9 ± 0.7	35 ± 3	26 ± 2	5.4 ± 0.2	22 ± 1
Quercetin-3-O- arabinoside	33 ± 3	22.2 ± 0.1	7.1 ± 0.5	33 ± 4	52 ± 4	30 ± 2	28 ± 4	59 ± 2	46 ± 3	13.9 ± 0.7	39 ± 2
Σ	**39 ± 3**	**30.6 ± 0.1**	**12.2 ± 0.5**	**56 ± 4**	**89 ± 5**	**47 ± 2**	**41 ± 4**	**94 ± 4**	**72 ± 4**	**19.3 ± 0.7**	**62 ± 3**
**N-phenylpropenoyl-L-aminoacids**											
N-Coumaroyl-L-aspartate 1	3.9 ± 0.2	10.5 ± 0.2	7.5 ± 0.5	6.2 ± 0.4	9.4 ± 0.5	5.6 ± 0.3	9.2 ± 0.6	6.4 ± 0.4	9.3 ± 0.7	9.9 ± 0.5	6.1 ± 0.5
N-Caffeoyl-L-aspartate	24 ± 1	62 ± 2	14 ± 1	153 ± 7	409 ± 7	123 ± 9	102 ± 9	371 ± 10	295 ± 26	37 ± 2	211 ± 20
N-Coumaroyl-L-aspartate 2	5.6 ± 0.4	20.6 ± 0.9	4.70 ± 0.01	46 ± 4	104 ± 9	52 ± 4	33 ± 2	149 ± 10	124 ± 7	14.5 ± 0.8	148 ± 14
N-Coumaroyl-L-glutamate	4.0 ± 0.3	4.0 ± 0.1	1.1 ± 0.1	3.4 ± 0.2	6.9 ± 0.6	6.8 ± 0.1	3.7 ± 0.2	10 ± 1	9.2 ± 0.6	3.1 ± 0.1	5.4 ± 0.6
N-coumaroyl-L-tyrosine	3.1 ± 0.3	8.6 ± 0.4	2.2 ± 0.1	12 ± 1	14.9 ± 0.5	10.3 ± 0.3	13 ± 2	49 ± 2	22 ± 2	3.2 ± 0.1	24 ± 2
Σ	**41 ± 1**	**105 ± 2**	**29 ± 1**	**220 ± 8**	**543 ± 12**	**197 ± 10**	**161 ± 9**	**584 ± 15**	**459 ± 27**	**68 ± 2**	**395 ± 24**
**Total polyphenols**	**493 ± 13**	**874 ± 11**	**238 ± 6**	**1686 ± 37**	**3108 ± 56**	**1040 ± 33**	**1244 ± 29**	**3134 ± 57**	**1831 ± 41**	**975 ± 29**	**1762 ± 43**

**METHYLXANTHINES**	**Concentration (g kg-1 of CBS)**										
Theobromine	3.4 ± 0.2	6.5 ± 0.1	3.0 ± 0.3	6.1 ± 0.4	8.2 ± 0.1	7.2 ± 0.4	7.5 ± 0.2	9.0 ± 0.1	7.4 ± 0.3	2.4 ± 0.2	8.5 ± 0.1
Caffeine	0.69 ± 0.04	0.74 ± 0.01	0.163 ± 0.003	1.2 ± 0.1	2.57 ± 0.05	1.2 ± 0.1	0.94 ± 0.03	3.3 ± 0.1	1.9 ± 0.2	0.51 ± 0.04	2.6 ± 0.1
**Total Methylxantines**	**4.1 ± 0.2**	**7.3 ± 0.1**	**3.2 ± 0.3**	**7.3 ± 0.4**	**10.7 ± 0.1**	**8.4 ± 0.4**	**8.4 ± 0.2**	**12.3 ± 0.1**	**9.3 ± 0.4**	**2.9 ± 0.2**	**11.1 ± 0.2**

Table S1 (continuation). Content of the single identified polyphenolic compounds and methylxantines in CBS powders from different geographical origins and cultivars. Data are presented as the mean (n=4) ± standard deviation.

**POLYPHENOLS**	**Concentration (mg kg-1 of CBS)**										
	ECU1	ECU2	ECU3	ECU4	ECU5	ECU6	ECU7	GHA	IVC	JAM	MAD
**Phenolic acids**											
Protocatechuic acid	99 ± 9	122 ± 9	206 ± 19	145 ± 2	113 ± 8	88 ± 5	108 ± 3	20.9 ± 0.8	54 ± 4	187 ± 18	87 ± 4
Σ	**99 ± 9**	**122 ± 9**	**206 ± 19**	**145 ± 2**	**113 ± 8**	**88 ± 5**	**108 ± 3**	**20.9 ± 0.8**	**54 ± 4**	**187 ± 18**	**87 ± 4**
**Flavan-3-ol**											
Catechin	46 ± 4	46 ± 2	114 ± 11	45 ± 3	48 ± 1	88 ± 5	180 ± 2	12.7 ± 0.5	27 ± 2	85 ± 3	139 ± 10
Epicatechin	203 ± 20	168 ± 7	366 ± 7	368 ± 13	239.29 ± 0.04	374 ± 6	256 ± 13	4.5 ± 0.3	50 ± 7	161 ± 16	749 ± 62
Σ	**250 ± 20**	**214 ± 8**	**480 ± 13**	**414 ± 14**	**287 ± 1**	**462 ± 8**	**437 ± 13**	**17.2 ± 0.6**	**77 ± 7**	**245 ± 16**	**888 ± 63**
**Catechin-3-O-glycosides**											
Catechin-3-O-glucoside	25 ± 2	12.1 ± 0.7	48 ± 1	22 ± 1	27.2 ± 0.1	15.6 ± 0.2	37 ± 3	6.8 ± 0.1	21 ± 1	58 ± 2	31.5 ± 0.6
Σ	**25 ± 2**	**12.1 ± 0.7**	**48 ± 1**	**22 ± 1**	**27.2 ± 0.1**	**15.6 ± 0.2**	**37 ± 3**	**6.8 ± 0.1**	**21 ± 1**	**58 ± 2**	**31.5 ± 0.6**
**Procyanidins B-type (PCB)**											
PCB	310 ± 23	167 ± 14	233 ± 12	198 ± 10	96 ± 8	277 ± 28	52 ± 2	6.8 ± 0.4	46 ± 5	57 ± 6	478 ± 19
Σ	**310 ± 23**	**167 ± 14**	**233 ± 12**	**198 ± 10**	**96 ± 8**	**277 ± 28**	**52 ± 2**	**6.8 ± 0.4**	**46 ± 5**	**57 ± 6**	**478 ± 19**
**Procyanidins B-type (PCB) trimers**											
PCB trimer 1	31 ± 1	18 ± 2	82 ± 5	39.7 ± 0.7	50 ± 5	33 ± 2	65 ± 3	2.5 ± 0.2	16 ± 1	131 ± 9	51.5 ± 0.9
PCB trimer 2	8.1 ± 0.5	3.3 ± 0.3	15.7 ± 0.5	2.6 ± 0.1	0.0 ± 0.0	24 ± 2	41 ± 3	0.5 ± 0.1	2.3 ± 0.2	163 ± 12	20 ± 2
PCB trimer 3	10.9 ± 0.9	5.7 ± 0.6	23.2 ± 0.7	14 ± 1	2.6 ± 0.1	11.9 ± 0.9	9.6 ± 0.5	3.6 ± 0.3	3.5 ± 0.4	8.4 ± 0.9	20 ± 2
PCB trimer 4	82 ± 7	73 ± 5	122 ± 10	124 ± 5	122 ± 13	112 ± 12	95 ± 3	7.6 ± 0.7	25 ± 3	67 ± 8	169 ± 13
PCB trimer 5	49 ± 6	50 ± 7	140 ± 6	51 ± 2	9.2 ± 0.9	73 ± 7	24 ± 2	6.5 ± 0.8	9.1 ± 0.8	46 ± 4	183 ± 14
PCB trimer 6	9 ± 1	0.0 ± 0.0	9.7 ± 0.9	12.3 ± 0.5	11.9 ± 0.7	4.3 ± 0.5	2.65 ± 0.02	2.1 ± 0.2	2.2 ± 0.3	7.3 ± 0.4	9.9 ± 0.3
PCB trimer 7	25.6 ± 0.9	11 ± 1	84 ± 1	4.7 ± 0.5	19.2 ± 0.8	39 ± 1	47 ± 4	3.4 ± 0.3	16 ± 1	32 ± 1	65 ± 4
Σ	**216 ± 9**	**162 ± 9**	**476 ± 13**	**248 ± 5**	**214 ± 14**	**298 ± 14**	**284 ± 7**	**26 ± 1**	**74 ± 3**	**455 ± 17**	**518 ± 20**
**Procyanidins A-type (PCA) glycosides**											
PCA hexoside 1	222 ± 31	87 ± 4	334 ± 20	172 ± 5	183 ± 7	136 ± 8	159 ± 12	8.5 ± 0.4	43.2 ± 0.8	171 ± 16	308 ± 8
PCA pentoside 1	29 ± 2	12.7 ± 0.9	47 ± 6	99 ± 11	31.98 ± 0.03	199 ± 16	28 ± 2	1.3 ± 0.1	2.6 ± 0.3	39 ± 3	30.1 ± 0.5
PCA trimer arabinoside	23 ± 3	14.1 ± 0.7	65 ± 3	7.5 ± 0.2	46 ± 3	13 ± 1	43 ± 3	3.4 ± 0.4	14 ± 2	39 ± 3	48 ± 4
PCA hexoside 2	20 ± 2	8.4 ± 0.8	40 ± 3	23 ± 2	25 ± 3	17 ± 2	40.1 ± 0.8	2.5 ± 0.1	5.9 ± 0.7	25 ± 3	18 ± 1
PCA pentoside 2	7.2 ± 0.4	n.d.	10 ± 1	5.5 ± 0.3	6.6 ± 0.8	5.8 ± 0.3	22.3 ± 0.9	0.65 ± 0.03	2.5 ± 0.3	13 ± 1	7.3 ± 0.2
Σ	**302 ± 31**	**122 ± 4**	**496 ± 22**	**306 ± 12**	**294 ± 8**	**371 ± 18**	**292 ± 12**	**16.3 ± 0.6**	**68 ± 2**	**287 ± 16**	**412 ± 9**
**Flavonols**											
Quercetin	2.6 ± 0.1	3.1 ± 0.3	9.9 ± 0.9	6.9 ± 0.4	3.0 ± 0.3	6.1 ± 0.6	2.8 ± 0.2	1.29 ± 0.04	1.1 ± 0.1	6.8 ± 0.9	6.9 ± 0.1
Σ	**2.6 ± 0.1**	**3.1 ± 0.3**	**9.9 ± 0.9**	**6.9 ± 0.4**	**3.0 ± 0.3**	**6.1 ± 0.6**	**2.8 ± 0.2**	**1.3 ± 0.0**	**1.1 ± 0.1**	**6.8 ± 0.9**	**6.9 ± 0.1**
**Flavonol-3-O-glycosides**											
Quercetin-3-O-glucoside	14.1 ± 0.9	8.8 ± 0.4	48 ± 2	21.49 ± 0.04	14 ± 2	16 ± 1	21.2 ± 0.7	3.5 ± 0.4	5.3 ± 0.3	23.1 ± 0.6	23 ± 1
Quercetin-3-O- arabinoside	21 ± 2	15.0 ± 0.4	64 ± 1	35.1 ± 0.7	23.0 ± 0.1	20 ± 1	44.2 ± 0.4	3.8 ± 0.2	8.1 ± 0.7	53.2 ± 0.8	27.2 ± 0.8
Σ	**35 ± 2**	**23.8 ± 0.6**	**112 ± 2**	**56.6 ± 0.7**	**37 ± 2**	**36 ± 2**	**65.4 ± 0.8**	**7.2 ± 0.4**	**13.4 ± 0.8**	**76 ± 1**	**50 ± 2**
**N-phenylpropenoyl-L-aminoacids**											
N-Coumaroyl-L-aspartate 1	12.8 ± 0.9	8.6 ± 0.5	14.1 ± 0.7	8.2 ± 0.7	13.4 ± 0.1	5.4 ± 0.3	22 ± 1	3.7 ± 0.3	6.4 ± 0.6	25 ± 2	5.2 ± 0.1
N-Caffeoyl-L-aspartate	102 ± 7	50 ± 3	431 ± 32	124 ± 6	57 ± 2	180 ± 8	138 ± 5	5.9 ± 0.4	22 ± 2	256 ± 17	269 ± 2
N-Coumaroyl-L-aspartate 2	25 ± 3	26 ± 3	184 ± 12	66 ± 3	65.8 ± 0.5	64 ± 6	94 ± 2	4.5 ± 0.5	11 ± 1	133 ± 3	75 ± 3
N-Coumaroyl-L-glutamate	4.8 ± 0.4	2.5 ± 0.3	8.5 ± 0.9	3.40 ± 0.03	5.8 ± 0.3	7.2 ± 0.7	5.3 ± 0.6	1.1 ± 0.1	4.2 ± 0.4	7.8 ± 0.4	5.7 ± 0.7
N-coumaroyl-L-tyrosine	12 ± 1	5.7 ± 0.8	29 ± 2	11 ± 2	17.8 ± 0.6	9 ± 1	17.5 ± 0.2	2.0 ± 0.2	6.5 ± 0.8	29 ± 1	17 ± 1
Σ	**155 ± 8**	**93 ± 4**	**667 ± 34**	**213 ± 7**	**160 ± 2**	**264 ± 10**	**277 ± 6**	**17.1 ± 0.7**	**50 ± 2**	**450 ± 17**	**371 ± 4**
**Total polyphenols**	**1394 ± 46**	**919 ± 21**	**2728 ± 50**	**1610 ± 23**	**1231 ± 20**	**1818 ± 39**	**1556 ± 20**	**120 ± 2**	**404 ± 10**	**1822 ± 39**	**2843 ± 70**

**METHYLXANTHINES**	**Concentration (g kg-1 of CBS)**										
Theobromine	5.9 ± 0.5	6.0 ± 0.3	8.6 ± 0.3	7.4 ± 0.1	6.3 ± 0.1	6.9 ± 0.3	8.0 ± 0.7	0.8 ± 0.1	3.5 ± 0.3	8.9 ± 0.2	8.25 ± 0.02
Caffeine	0.95 ± 0.04	1.15 ± 0.03	2.1 ± 0.1	1.7 ± 0.1	1.7 ± 0.1	1.2 ± 0.1	2.8 ± 0.3	0.151 ± 0.004	0.67 ± 0.04	3.00 ± 0.02	2.29 ± 0.01
**Total Methylxantines**	**6.8 ± 0.5**	**7.2 ± 0.3**	**10.6 ± 0.3**	**9.1 ± 0.2**	**8.0 ± 0.1**	**8.1 ± 0.3**	**10.8 ± 0.8**	**0.9 ± 0.1**	**4.2 ± 0.3**	**11.9 ± 0.2**	**10.55 ± 0.02**

n.d. = not detected

Table S1 (continuation). Content of the single identified polyphenolic compounds and methylxantines in CBS powders from different geographical origins and cultivars. Data are presented as the mean (n=4) ± standard deviation.

POLYPHENOLS	Concentration (mg kg-1 of CBS)										
	MEX	PER1	PER2	SAT1	SAT2	SAT3	SLE	TAN	TOG1	TOG2	UGA1
**Phenolic acids**											
Protocatechuic acid	241 ± 6	120 ± 7	87 ± 3	120 ± 4	198 ± 11	164 ± 16	14.4 ± 0.7	88 ± 7	14 ± 1	16 ± 1	57 ± 3
Σ	**241 ± 6**	**120 ± 7**	**87 ± 3**	**120 ± 4**	**198 ± 11**	**164 ± 16**	**14.4 ± 0.7**	**88 ± 7**	**14 ± 1**	**16 ± 1**	**57 ± 3**
**Flavan-3-ol**											
Catechin	93 ± 5	61 ± 4	85 ± 3	26.4 ± 0.8	120 ± 9	114 ± 7	35 ± 3	35 ± 2	19 ± 1	20 ± 1	14 ± 1
Epicatechin	430 ± 12	352 ± 30	300 ± 42	325 ± 29	632 ± 18	542 ± 45	191 ± 14	137 ± 17	25 ± 3	37 ± 2	31 ± 2
Σ	**523 ± 13**	**413 ± 30**	**385 ± 42**	**352 ± 29**	**751 ± 20**	**656 ± 46**	**225 ± 14**	**173 ± 18**	**43 ± 3**	**58 ± 2**	**45 ± 3**
**Catechin-3-O-glycosides**											
Catechin-3-O-glucoside	89 ± 4	27 ± 2	33 ± 2	42 ± 1	101 ± 3	79 ± 3	29 ± 2	17.9 ± 0.7	27 ± 2	28 ± 2	6.4 ± 0.3
Σ	**89 ± 4**	**27 ± 2**	**33 ± 2**	**42 ± 1**	**101 ± 3**	**79 ± 3**	**29 ± 2**	**17.9 ± 0.7**	**27 ± 2**	**28 ± 2**	**6.4 ± 0.3**
**Procyanidins B-type (PCB)**											
PCB	261 ± 35	138 ± 2	341 ± 26	640 ± 41	191 ± 20	246 ± 30	136 ± 12	253 ± 18	136 ± 12	146 ± 14	129 ± 5
Σ	**261 ± 35**	**138 ± 2**	**341 ± 26**	**640 ± 41**	**191 ± 20**	**246 ± 30**	**136 ± 12**	**253 ± 18**	**136 ± 12**	**146 ± 14**	**129 ± 5**
**Procyanidins B-type (PCB) trimers**											
PCB trimer 1	106 ± 3	42 ± 1	51 ± 5	24 ± 3	96 ± 6	65 ± 7	2.7 ± 0.1	24 ± 2	12.0 ± 0.6	11 ± 1	10 ± 1
PCB trimer 2	19 ± 1	1.5 ± 0.1	12 ± 1	35 ± 3	3.5 ± 0.3	5.9 ± 0.4	4.7 ± 0.4	7.6 ± 0.7	17.3 ± 0.8	13.0 ± 0.6	n.d.
PCB trimer 3	12 ± 1	10 ± 1	9.1 ± 0.7	40 ± 5	11 ± 1	10.8 ± 0.8	1.5 ± 0.1	9 ± 1	4.0 ± 0.4	12 ± 1	3.1 ± 0.4
PCB trimer 4	128 ± 17	124 ± 8	159 ± 11	143 ± 15	264 ± 31	242 ± 30	73 ± 1	130 ± 16	14.9 ± 0.7	17 ± 2	31 ± 4
PCB trimer 5	163 ± 14	73 ± 6	56 ± 6	223 ± 11	176 ± 17	132 ± 3	71 ± 9	51 ± 6	74 ± 7	45 ± 3	21 ± 2
PCB trimer 6	4.7 ± 0.3	6.2 ± 0.6	16 ± 2	2.3 ± 0.2	14 ± 1	21 ± 2	8.1 ± 0.4	20 ± 1	7.4 ± 0.4	4.4 ± 0.6	3.3 ± 0.1
PCB trimer 7	104 ± 6	16 ± 1	37 ± 3	16.0 ± 0.9	89 ± 5	89 ± 11	38 ± 5	21 ± 3	25 ± 3	22 ± 2	7.1 ± 0.6
Σ	**538 ± 23**	**272 ± 10**	**340 ± 14**	**484 ± 20**	**654 ± 36**	**566 ± 32**	**199 ± 11**	**263 ± 17**	**154 ± 7**	**123 ± 5**	**76 ± 5**
**Procyanidins A-type (PCA) glycosides**											
PCA hexoside 1	417 ± 37	302 ± 19	236 ± 6	199 ± 19	561 ± 17	479 ± 60	86 ± 10	126 ± 16	20 ± 2	21.5 ± 0.8	30 ± 3
PCA pentoside 1	49 ± 7	31 ± 2	46 ± 4	103 ± 8	75 ± 7	43 ± 6	3.1 ± 0.1	24 ± 2	10.3 ± 0.7	21 ± 2	3.0 ± 0.2
PCA trimer arabinoside	114 ± 4	25 ± 2	32 ± 3	43 ± 2	84 ± 4	65 ± 3	13 ± 1	24 ± 1	22.0 ± 0.4	22.0 ± 0.9	5.8 ± 0.4
PCA hexoside 2	46 ± 4	46 ± 3	16 ± 2	35 ± 2	73 ± 7	71 ± 9	20 ± 2	12 ± 1	12.8 ± 0.9	19 ± 2	4.5 ± 0.3
PCA pentoside 2	31 ± 4	14.2 ± 0.7	21 ± 2	7.4 ± 0.3	40 ± 4	24 ± 3	7.2 ± 0.7	14 ± 1	7 ± 1	2.4 ± 0.3	1.6 ± 0.1
Σ	**658 ± 38**	**417 ± 20**	**351 ± 8**	**387 ± 21**	**833 ± 21**	**682 ± 61**	**129 ± 10**	**199 ± 16**	**73 ± 3**	**85 ± 3**	**45 ± 3**
**Flavonols**											
Quercetin	22.4 ± 0.6	7.5 ± 0.3	6.3 ± 0.5	4.7 ± 0.3	13.9 ± 0.5	8.0 ± 0.9	2.0 ± 0.1	2.9 ± 0.2	1.1 ± 0.1	1.4 ± 0.1	2.0 ± 0.1
Σ	**22.4 ± 0.6**	**7.5 ± 0.3**	**6.3 ± 0.5**	**4.7 ± 0.3**	**13.9 ± 0.5**	**8.0 ± 0.9**	**2.0 ± 0.1**	**2.9 ± 0.2**	**1.1 ± 0.1**	**1.4 ± 0.1**	**2.0 ± 0.1**
**Flavonol-3-O-glycosides**											
Quercetin-3-O-glucoside	23 ± 1	18 ± 1	19.8 ± 0.2	13.1 ± 0.5	35 ± 3	21 ± 1	6.9 ± 0.2	9.3 ± 0.9	4.8 ± 0.5	4.1 ± 0.4	5.9 ± 0.7
Quercetin-3-O- arabinoside	70 ± 4	36.6 ± 0.8	32.2 ± 0.6	25 ± 2	87 ± 5	42 ± 1	21 ± 1	23 ± 1	20 ± 1	20 ± 2	10.4 ± 0.1
Σ	**93 ± 4**	**55 ± 1**	**51.9 ± 0.7**	**38 ± 2**	**122 ± 6**	**62 ± 2**	**28 ± 1**	**32 ± 2**	**24 ± 1**	**24 ± 2**	**16.3 ± 0.7**
**N-phenylpropenoyl-L-aminoacids**											
N-Coumaroyl-L-aspartate 1	14 ± 1	11.1 ± 0.7	7.1 ± 0.7	9.4 ± 0.8	12 ± 1	14.8 ± 0.5	4.6 ± 0.4	6.9 ± 0.6	3.1 ± 0.2	2.8 ± 0.2	4.4 ± 0.4
N-Caffeoyl-L-aspartate	653 ± 40	195 ± 16	248 ± 6	141 ± 13	358 ± 29	244 ± 3	37 ± 1	50 ± 4	34 ± 2	23 ± 2	34 ± 2
N-Coumaroyl-L-aspartate 2	94 ± 3	85 ± 3	122 ± 8	29 ± 2	118 ± 8	94 ± 11	9.4 ± 0.2	23.5 ± 0.9	7.2 ± 0.1	5.4 ± 0.3	14.9 ± 0.8
N-Coumaroyl-L-glutamate	9.4 ± 0.2	6.2 ± 0.6	8.4 ± 0.4	7.4 ± 0.8	12.6 ± 0.4	11 ± 1	3.2 ± 0.1	5.1 ± 0.2	9.3 ± 0.7	9.1 ± 0.9	3.1 ± 0.2
N-coumaroyl-L-tyrosine	40 ± 2	13 ± 2	18 ± 1	16 ± 1	38 ± 3	42 ± 1	6.9 ± 0.6	7.3 ± 0.4	7.3 ± 0.6	4.5 ± 0.4	4.9 ± 0.2
Σ	**810 ± 40**	**311 ± 16**	**404 ± 10**	**203 ± 13**	**539 ± 31**	**406 ± 11**	**61 ± 1**	**93 ± 4**	**61 ± 2**	**45 ± 2**	**61 ± 2**
**Total polyphenols**	**3235 ± 71**	**1759 ± 41**	**1999 ± 53**	**2271 ± 59**	**3403 ± 61**	**2869 ± 90**	**823 ± 24**	**1123 ± 35**	**534 ± 15**	**526 ± 16**	**438 ± 9**

METHYLXANTHINES	Concentration (g kg-1 of CBS)										
Theobromine	8.5 ± 0.3	6.8 ± 0.4	8.1 ± 0.7	7.4 ± 0.3	8.7 ± 0.2	8.5 ± 0.3	4.4 ± 0.3	6.9 ± 0.5	4.4 ± 0.3	3.7 ± 0.1	4.0 ± 0.4
Caffeine	1.3 ± 0.1	1.5 ± 0.1	1.6 ± 0.1	1.46 ± 0.05	1.4 ± 0.1	1.49 ± 0.04	0.38 ± 0.03	1.1 ± 0.1	0.40 ± 0.04	0.35 ± 0.02	0.50 ± 0.03
**Total Methylxantines**	**9.7 ± 0.3**	**8.3 ± 0.4**	**9.7 ± 0.7**	**8.9 ± 0.3**	**10.1 ± 0.2**	**10.0 ± 0.3**	**4.7 ± 0.3**	**8.0 ± 0.5**	**4.8 ± 0.3**	**4.1 ± 0.1**	**4.5 ± 0.4**

n.d. = not detected

Table S1 (continuation). Content of the single identified polyphenolic compounds and methylxantines in CBS powders from different geographical origins and cultivars. Data are presented as the mean (n=4) ± standard deviation.

**POLYPHENOLS**	**Concentration (mg kg-1 of CBS)**										
	UGA2	VEN1	VEN2	VEN3	VEN4	VEN5	VEN6	VEN7	VEN8	VEN9	VEN10
**Phenolic acids**											
Protocatechuic acid	108 ± 8	119 ± 5	73 ± 2	108 ± 4	46 ± 3	121 ± 9	124 ± 9	104 ± 4	136 ± 4	86 ± 5	12.05 ± 0.02
Σ	**108 ± 8**	**119 ± 5**	**73 ± 2**	**108 ± 4**	**46 ± 3**	**121 ± 9**	**124 ± 9**	**104 ± 4**	**136 ± 4**	**86 ± 5**	**12.05 ± 0.02**
**Flavan-3-ol**											
Catechin	51 ± 5	21 ± 2	35 ± 2	73 ± 4	53 ± 2	56 ± 3	66 ± 3	133 ± 5	118 ± 6	127 ± 5	77 ± 2
Epicatechin	16 ± 1	294 ± 26	159 ± 19	340 ± 31	118 ± 5	108 ± 12	190 ± 16	186 ± 1	229 ± 4	88 ± 5	229.23 ± 0.05
Σ	**67 ± 5**	**314 ± 26**	**195 ± 19**	**412 ± 31**	**171 ± 5**	**164 ± 12**	**256 ± 16**	**319 ± 5**	**347 ± 7**	**215 ± 7**	**306 ± 2**
**Catechin-3-O-glycosides**											
Catechin-3-O-glucoside	13 ± 1	17 ± 1	14 ± 1	52 ± 5	20 ± 1	25 ± 1	30 ± 2	34.8 ± 0.5	50 ± 3	25 ± 2	31.9 ± 0.6
Σ	**13 ± 1**	**17 ± 1**	**14 ± 1**	**52 ± 5**	**20 ± 1**	**25 ± 1**	**30 ± 2**	**34.8 ± 0.5**	**50 ± 3**	**25 ± 2**	**31.9 ± 0.6**
**Procyanidins B-type (PCB)**											
PCB	38 ± 5	408 ± 53	673 ± 52	78 ± 4	83 ± 3	55 ± 3	182 ± 10	106 ± 9	54 ± 2	68 ± 5	104 ± 12
Σ	**38 ± 5**	**408 ± 53**	**673 ± 52**	**78 ± 4**	**83 ± 3**	**55 ± 3**	**182 ± 10**	**106 ± 9**	**54 ± 2**	**68 ± 5**	**104 ± 12**
**Procyanidins B-type (PCB) trimers**											
PCB trimer 1	14 ± 1	20 ± 1	70 ± 5	74 ± 2	16 ± 1	60 ± 5	27 ± 1	65.1 ± 0.2	92 ± 7	49 ± 5	16.5 ± 0.5
PCB trimer 2	6.7 ± 0.5	21 ± 2	73 ± 8	26 ± 1	13 ± 2	36 ± 3	23 ± 1	110 ± 3	48.4 ± 0.1	36 ± 3	1.5 ± 0.1
PCB trimer 3	14 ± 1	16 ± 1	97 ± 11	53 ± 3	17 ± 2	30 ± 2	18 ± 2	20 ± 2	10 ± 1	8.6 ± 0.7	15 ± 2
PCB trimer 4	18 ± 2	105 ± 10	101 ± 5	104 ± 10	30 ± 1	38 ± 1	99 ± 6	48 ± 5	62 ± 4	34.4 ± 0.1	123 ± 3
PCB trimer 5	13 ± 1	130 ± 9	260 ± 33	79 ± 3	42 ± 2	35 ± 4	168 ± 15	62 ± 5	22 ± 3	40 ± 4	27 ± 2
PCB trimer 6	3.7 ± 0.2	7.0 ± 0.8	1.6 ± 0.2	3.9 ± 0.4	0.8 ± 0.1	4.5 ± 0.2	2.4 ± 0.2	2.9 ± 0.1	3.63 ± 0.03	3.3 ± 0.4	8.6 ± 0.1
PCB trimer 7	28 ± 3	32 ± 2	13.8 ± 0.6	37 ± 4	6.9 ± 0.3	16 ± 1	38 ± 5	35.7 ± 0.3	47 ± 4	36 ± 4	29.5 ± 0.4
Σ	**97 ± 4**	**330 ± 14**	**617 ± 36**	**377 ± 12**	**126 ± 3**	**220 ± 7**	**375 ± 17**	**342 ± 8**	**286 ± 10**	**208 ± 8**	**220 ± 4**
**Procyanidins A-type (PCA) glycosides**											
PCA hexoside 1	34 ± 4	172 ± 8	34 ± 1	315 ± 14	56 ± 3	62 ± 2	131 ± 15	181.2 ± 0.3	211 ± 21	158 ± 14	160 ± 11
PCA pentoside 1	3.0 ± 0.4	34 ± 4	98 ± 4	65 ± 4	13 ± 2	15.1 ± 0.6	45 ± 4	29 ± 2	37.5 ± 0.6	22 ± 2	11 ± 1
PCA trimer arabinoside	7.6 ± 0.3	32 ± 3	24 ± 3	34 ± 4	16 ± 1	25 ± 2	37 ± 3	25.8 ± 0.2	36 ± 1	26.8 ± 0.1	18 ± 2
PCA hexoside 2	2.7 ± 0.3	13 ± 1	20 ± 2	30 ± 2	9.2 ± 0.6	20 ± 2	29 ± 2	14.9 ± 0.4	29 ± 2	12 ± 2	11.5 ± 0.1
PCA pentoside 2	0.91 ± 0.05	8.3 ± 0.7	2.3 ± 0.2	22 ± 3	4.8 ± 0.3	5.5 ± 0.7	9.3 ± 0.1	16 ± 2	16 ± 1	10.3 ± 0.6	8.6 ± 0.2
Σ	**48 ± 4**	**259 ± 10**	**179 ± 6**	**467 ± 16**	**98 ± 4**	**127 ± 3**	**252 ± 16**	**267 ± 3**	**329 ± 21**	**230 ± 14**	**209 ± 11**
**Flavonols**											
Quercetin	2.4 ± 0.2	3.2 ± 0.3	5.9 ± 0.8	3.4 ± 0.3	2.6 ± 0.1	3.3 ± 0.3	6.1 ± 0.5	1.63 ± 0.02	2.94 ± 0.04	3.5 ± 0.4	2.4 ± 0.1
Σ	**2.4 ± 0.2**	**3.2 ± 0.3**	**5.9 ± 0.8**	**3.4 ± 0.3**	**2.6 ± 0.1**	**3.3 ± 0.3**	**6.1 ± 0.5**	**1.63 ± 0.02**	**2.94 ± 0.04**	**3.5 ± 0.4**	**2.4 ± 0.1**
**Flavonol-3-O-glycosides**											
Quercetin-3-O-glucoside	5.1 ± 0.4	10.4 ± 0.8	18.2 ± 0.6	25 ± 2	7.1 ± 0.5	10.9 ± 0.6	20 ± 2	12 ± 1	21 ± 1	6.5 ± 0.2	10.6 ± 0.3
Quercetin-3-O- arabinoside	10.6 ± 0.3	17.5 ± 0.8	23 ± 2	51 ± 3	14.4 ± 0.4	22.1 ± 0.7	28.7 ± 0.7	14.9 ± 0.4	37.0 ± 0.8	17 ± 1	25 ± 1
Σ	**15.8 ± 0.5**	**28 ± 1**	**42 ± 2**	**76 ± 4**	**21.5 ± 0.7**	**33.0 ± 0.9**	**49 ± 2**	**27 ± 1**	**58 ± 1**	**24 ± 1**	**36 ± 1**
**N-phenylpropenoyl-L-aminoacids**											
N-Coumaroyl-L-aspartate 1	13 ± 1	5.4 ± 0.2	1.4 ± 0.1	21.3 ± 0.9	6.5 ± 0.3	19 ± 2	9.0 ± 0.6	8.69 ± 0.03	13.4 ± 0.4	5.8 ± 0.3	4.2 ± 0.3
N-Caffeoyl-L-aspartate	18 ± 1	60 ± 3	169 ± 16	194 ± 10	94 ± 9	98 ± 5	191 ± 17	200 ± 4	189 ± 4	90 ± 2	149 ± 6
N-Coumaroyl-L-aspartate 2	21 ± 1	23 ± 2	29 ± 2	75 ± 4	24 ± 1	55 ± 2	65 ± 3	92 ± 3	87 ± 3	62 ± 3	49 ± 3
N-Coumaroyl-L-glutamate	2.2 ± 0.2	5.7 ± 0.3	9.8 ± 0.6	5.2 ± 0.6	3.8 ± 0.5	3.4 ± 0.4	4.3 ± 0.4	2.4 ± 0.3	5.8 ± 0.7	7 ± 1	3.2 ± 0.3
N-coumaroyl-L-tyrosine	4.6 ± 0.4	10 ± 1	7.5 ± 0.5	23 ± 2	4.5 ± 0.2	13 ± 1	12.1 ± 0.9	18.8 ± 0.2	26.8 ± 0.5	13.9 ± 0.5	8 ± 1
Σ	**58 ± 2**	**104 ± 4**	**217 ± 16**	**319 ± 11**	**133 ± 9**	**188 ± 6**	**281 ± 17**	**322 ± 5**	**322 ± 5**	**178 ± 4**	**214 ± 7**
**Total polyphenols**	**449 ± 12**	**1583 ± 62**	**2015 ± 68**	**1892 ± 40**	**702 ± 13**	**937 ± 18**	**1555 ± 36**	**1523 ± 15**	**1584 ± 25**	**1036 ± 20**	**1136 ± 18**

**METHYLXANTHINES**	**Concentration (g kg-1 of CBS)**										
Theobromine	6.8 ± 0.3	6.0 ± 0.4	6.5 ± 0.4	8.2 ± 0.6	3.2 ± 0.1	6.0 ± 0.5	7.2 ± 0.7	7.74 ± 0.03	8.4 ± 0.2	6.9 ± 0.3	5.3 ± 0.2
Caffeine	0.99 ± 0.01	1.3 ± 0.1	2.2 ± 0.1	3.0 ± 0.1	1.21 ± 0.04	2.2 ± 0.2	2.6 ± 0.2	3.37 ± 0.03	3.0 ± 0.1	2.6 ± 0.1	1.9 ± 0.1
**Total Methylxantines**	**7.8 ± 0.3**	**7.2 ± 0.4**	**8.8 ± 0.4**	**11.2 ± 0.6**	**4.4 ± 0.1**	**8.3 ± 0.5**	**9.9 ± 0.7**	**11.11 ± 0.05**	**11.4 ± 0.2**	**9.6 ± 0.3**	**7.2 ± 0.2**

Table S2. Pairwise comparison of the phenolic compounds quantified in CBSs by RP-HPLC-PDA that could be used as potential geographical origin markers for the American and African continents.

Protocatechuic_acid	Africa
America	0.0023

N-Coumaroyl-L-aspartate_1	Africa
America	0.008

N-Caffeoyl-L-aspartate	Africa
America	3.10E-08

**Catechin**	Africa
America	3.40E-08

PCA_hexoside_1	Africa
America	9.70E-05

Catechin-3-O-glucoside	Africa
America	0.19

Quercetin	Africa
America	1.00E-05

**PCB_trimer_1**	Africa
America	3.30E-11

PCB_trimer_6	Africa
America	0.71

N-Coumaroyl-L-aspartate_2	Africa
America	2.60E-09

PCB_trimer_2	Africa
America	1.80E-06

PCB	Africa
America	0.046

Epicatechin	Africa
America	4.10E-06

N-Coumaroyl-L-glutamate	Africa
America	0.41

PCB_trimer_3	Africa
America	0.0098

PCB_trimer_4	Africa
America	0.0061

PCB_trimer_5	Africa
America	0.0069

PCA_pentoside_1	Africa
America	2.10E-07

PCB_trimer_7	Africa
America	0.0048

PCA_trimer_arabinoside	Africa
America	0.0046

PCA_hexoside_2	Africa
America	0.004

N-coumaroyl-L-tyrosine	Africa
America	0.00025

PCA_pentoside_2	Africa
America	0.005

Quercetin-3-O-glucoside	Africa
America	1.60E-08

Quercetin-3-O-arabinoside	Africa
America	1.90E-05

FDR (false discovery rate)<0.001; FDR<0.01 and FDR<0.05 are highlighted in green, yellow, and red, respectively. Compounds highlighted in bold were used to build the box plots that are displayed in the main

Table S3. Pairwise comparison of the phenolic compounds quantified in CBSs by RP-HPLC-PDA that could be used as potential geographical origin markers for different countries within the African continent.

Protocatechuic_acid

	CAM	CON	GHA	IVC	MAD	SAT	SLE	TAN	TOG
CON	0.02381	-	-	-	-	-	-	-	-
GHA	0.05357	0.02381	-	-	-	-	-	-	-
IVC	1	0.02381	0.05357	-	-	-	-	-	-
MAD	1	0.11285	0.17143	0.17143	-	-	-	-	-
SAT	0.00549	0.60038	0.00549	0.00549	0.04945	-	-	-	-
SLE	0.17143	0.11285	0.17143	0.17143	0.41667	0.04945	-	-	-
TAN	1	0.02381	0.05357	0.05357	1	0.00549	0.17143	-	-
TOG	0.01299	0.00549	0.01299	0.01299	0.07692	0.00036	1	0.01299	-
UGA	0.80861	0.00549	0.01299	0.11285	1	0.00036	0.07692	1	0.00233

N-Coumaroyl-L-aspartate_1

	CAM	CON	GHA	IVC	MAD	SAT	SLE	TAN	TOG
CON	0.56044	-	-	-	-	-	-	-	-
GHA	0.06767	0.03297	-	-	-	-	-	-	-
IVC	0.06767	0.44048	0.06767	-	-	-	-	-	-
MAD	0.17859	0.13393	0.17859	0.17859	-	-	-	-	-
SAT	0.11913	0.02133	0.00824	0.00824	0.06593	-	-	-	-
SLE	0.17859	0.13393	0.17859	0.17859	0.42857	0.06593	-	-	-
TAN	0.17859	0.56044	0.06767	0.56044	0.17859	0.00824	0.17859	-	-
TOG	0.0202	0.00824	0.0303	0.0202	0.09524	0.00071	0.09524	0.0202	-
UGA	1	1	0.0303	1	1	0.17859	0.8	1	0.0035

N-Caffeoyl-L-aspartate

	CAM	CON	GHA	IVC	MAD	SAT	SLE	TAN	TOG
CON	0.02521	-	-	-	-	-	-	-	-
GHA	0.05844	0.02521	-	-	-	-	-	-	-
IVC	1	0.02521	0.05844	-	-	-	-	-	-
MAD	0.16667	0.12363	0.16667	0.16667	-	-	-	-	-
SAT	0.00549	0.00162	0.00549	0.00549	0.65067	-	-	-	-
SLE	1	0.12363	0.16667	0.16667	0.40541	0.05495	-	-	-
TAN	1	0.02521	0.05844	0.05844	0.16667	0.00549	0.16667	-	-
TOG	1	0.00549	0.01399	0.16667	0.08333	0.00036	0.14286	0.01399	-
UGA	1	0.00549	0.01399	1	0.08333	0.00036	0.14286	0.01399	0.662

Catechin

	CAM	CON	GHA	IVC	MAD	SAT	SLE	TAN	TOG
CON	0.1875	-	-	-	-	-	-	-	-
GHA	0.12245	0.04762	-	-	-	-	-	-	-
IVC	0.08036	0.23377	0.08036	-	-	-	-	-	-
MAD	0.1875	0.13975	0.1875	0.1875	-	-	-	-	-
SAT	0.01648	0.03047	0.01648	0.1875	0.08036	-	-	-	-
SLE	0.1875	0.13975	0.1875	0.1875	0.44118	0.66825	-	-	-
TAN	0.08036	0.04762	0.08036	0.08036	0.1875	0.4739	0.90909	-	-
TOG	0.67464	0.44356	0.0303	0.0303	0.10526	0.00071	0.10526	0.0303	-
UGA	0.90909	0.93492	0.10909	1	0.10526	0.0558	1	1	1

PCA_hexoside_1

	CAM	CON	GHA	IVC	MAD	SAT	SLE	TAN	TOG
CON	0.16667	-	-	-	-	-	-	-	-
GHA	0.0559	0.02381	-	-	-	-	-	-	-
IVC	1	0.02381	0.0559	-	-	-	-	-	-
MAD	0.16667	0.1148	0.16667	0.16667	-	-	-	-	-
SAT	0.00495	0.00162	0.00495	0.00495	0.63398	-	-	-	-
SLE	1	0.72321	0.16667	0.16667	0.40541	0.05205	-	-	-
TAN	1	0.56391	0.0559	0.0559	0.16667	0.00495	0.16667	-	-
TOG	1	0.00495	0.01136	0.01136	0.07407	0.00036	0.07407	0.01136	-
UGA	1	0.00495	0.01136	0.01136	0.07407	0.00036	0.07407	0.01136	0.00175

Catechin-3-O-glucoside

	CAM	CON	GHA	IVC	MAD	SAT	SLE	TAN	TOG
CON	0.45306	-	-	-	-	-	-	-	-
GHA	0.06122	0.02857	-	-	-	-	-	-	-
IVC	1	0.57915	0.06122	-	-	-	-	-	-
MAD	0.63158	0.72321	0.19355	0.19355	-	-	-	-	-
SAT	0.00618	0.12524	0.00618	0.00618	0.05818	-	-	-	-
SLE	1	0.72321	0.19355	0.19355	0.45306	0.05818	-	-	-
TAN	1	0.57915	0.06122	0.06122	0.19355	0.00618	0.19355	-	-
TOG	1	0.45306	0.01299	0.01299	0.16	0.00036	0.375	0.01299	-
UGA	0.01299	0.00618	1	0.01299	0.08696	0.00036	0.08696	0.01299	0.00233

Quercetin

	CAM	CON	GHA	IVC	MAD	SAT	SLE	TAN	TOG
CON	0.08	-	-	-	-	-	-	-	-
GHA	1	0.02857	-	-	-	-	-	-	-
IVC	0.40602	0.02857	0.06767	-	-	-	-	-	-
MAD	0.17143	0.1148	0.17143	0.17143	-	-	-	-	-
SAT	0.00549	0.1148	0.00549	0.00549	0.76078	-	-	-	-
SLE	1	0.1148	0.17143	0.17143	0.40541	0.06181	-	-	-
TAN	1	0.08	0.06767	0.06767	0.17143	0.00549	0.17143	-	-
TOG	0.76818	0.00549	1	0.26768	0.08	0.00036	0.08	0.01399	-
UGA	1	0.00549	0.01399	0.01399	0.08	0.00036	0.08	0.01399	0.00233

PCB_trimer_1

	CAM	CON	GHA	IVC	MAD	SAT	SLE	TAN	TOG
CON	0.02857	-	-	-	-	-	-	-	-
GHA	1	0.02857	-	-	-	-	-	-	-
IVC	0.05844	0.52265	0.05844	-	-	-	-	-	-
MAD	0.15789	0.10909	0.15789	0.15789	-	-	-	-	-
SAT	0.00706	0.00162	0.00706	0.00706	0.5887	-	-	-	-
SLE	1	0.10909	0.30769	0.15789	0.375	0.05818	-	-	-
TAN	0.05844	0.02857	0.05844	0.05844	0.15789	0.14526	0.15789	-	-
TOG	0.10909	0.00706	0.01515	0.01515	0.07692	0.00036	0.07692	0.01515	-
UGA	0.10909	0.11795	0.01515	0.04545	0.07692	0.00036	0.07692	0.01515	0.67539

PCB_trimer_6

	CAM	CON	GHA	IVC	MAD	SAT	SLE	TAN	TOG
CON	1	-	-	-	-	-	-	-	-
GHA	1	0.612	-	-	-	-	-	-	-
IVC	1	0.612	1	-	-	-	-	-	-
MAD	0.207	0.161	0.207	0.207	-	-	-	-	-
SAT	0.154	0.161	0.066	0.125	0.651	-	-	-	-
SLE	0.651	0.161	0.207	0.207	0.469	0.651	-	-	-
TAN	0.107	0.054	0.107	0.107	0.207	0.207	0.207	-	-
TOG	0.909	0.661	0.026	0.026	0.125	0.345	0.19	0.026	-
UGA	1	0.47	0.026	0.026	0.125	0.345	0.125	0.026	0.026

N-Coumaroyl-L-aspartate_2

	CAM	CON	GHA	IVC	MAD	SAT	SLE	TAN	TOG
CON	0.02381	-	-	-	-	-	-	-	-
GHA	0.16216	0.02381	-	-	-	-	-	-	-
IVC	1	0.02381	0.05357	-	-	-	-	-	-
MAD	0.16216	0.10714	0.16216	0.16216	-	-	-	-	-
SAT	0.00549	0.33551	0.00549	0.00549	0.5887	-	-	-	-
SLE	1	0.10714	0.16216	0.31579	0.375	0.04945	-	-	-
TAN	0.05357	0.02381	0.05357	0.05357	0.16216	0.00549	0.16216	-	-
TOG	1	0.00549	0.01299	0.01299	0.07143	0.00036	0.07143	0.01299	-
UGA	0.50554	0.00549	0.01299	0.01299	0.07143	0.00036	0.07143	0.03828	0.00233

PCB_trimer_2

	CAM	CON	GHA	IVC	MAD	SAT	SLE	TAN	TOG
CON	0.039	-	-	-	-	-	-	-	-
GHA	0.071	0.039	-	-	-	-	-	-	-
IVC	0.071	0.039	0.071	-	-	-	-	-	-
MAD	0.188	0.14	0.188	0.188	-	-	-	-	-
SAT	0.146	0.836	0.012	0.012	0.634	-	-	-	-
SLE	0.188	0.367	0.188	0.188	0.417	0.634	-	-	-
TAN	0.071	0.836	0.071	0.071	0.188	0.461	0.188	-	-
TOG	0.023	0.012	0.023	0.023	0.091	0.315	0.091	0.023	-
UGA	1	0.091	1	1	0.091	0.239	1	0.071	0.012

PCB

	CAM	CON	GHA	IVC	MAD	SAT	SLE	TAN	TOG
CON	0.02857	-	-	-	-	-	-	-	-
GHA	0.05844	0.02857	-	-	-	-	-	-	-
IVC	1	0.02857	0.05844	-	-	-	-	-	-
MAD	0.17647	0.12363	0.17647	0.17647	-	-	-	-	-
SAT	0.00989	0.08	0.00989	0.00989	0.5887	-	-	-	-
SLE	0.17647	0.48214	0.17647	0.17647	0.39474	0.05818	-	-	-
TAN	0.05844	0.32143	0.05844	0.05844	0.17647	0.9744	0.17647	-	-
TOG	0.01653	0.47722	0.01653	0.01653	0.08	0.00036	0.58537	0.01653	-
UGA	0.27532	0.05619	0.01653	0.84567	0.08	0.00036	0.32432	0.01653	0.01748

Epicatechin

	CAM	CON	GHA	IVC	MAD	SAT	SLE	TAN	TOG
CON	0.14286	-	-	-	-	-	-	-	-
GHA	0.04945	0.02256	-	-	-	-	-	-	-
IVC	0.7013	0.02256	0.04945	-	-	-	-	-	-
MAD	0.14286	0.10045	0.14286	0.14286	-	-	-	-	-
SAT	0.00549	0.00162	0.00549	0.00549	0.0471	-	-	-	-
SLE	0.14286	0.10045	0.14286	0.14286	0.34884	0.0471	-	-	-
TAN	0.04945	0.7619	0.04945	0.04945	0.14286	0.00549	0.14286	-	-
TOG	0.02139	0.00549	0.01136	0.01136	0.06667	0.00036	0.06667	0.01136	-
UGA	0.01136	0.00549	0.01136	0.01136	0.06667	0.00036	0.06667	0.01136	0.14286

N-Coumaroyl-L-glutamate

	CAM	CON	GHA	IVC	MAD	SAT	SLE	TAN	TOG
CON	0.40659	-	-	-	-	-	-	-	-
GHA	0.25	0.02521	-	-	-	-	-	-	-
IVC	0.17647	0.95681	0.0559	-	-	-	-	-	-
MAD	0.17647	0.68878	0.17647	0.17647	-	-	-	-	-
SAT	0.00618	0.00618	0.00618	0.00618	0.05205	-	-	-	-
SLE	1	0.11905	0.17647	0.17647	0.39474	0.05205	-	-	-
TAN	0.0559	0.52265	0.0559	0.0559	0.32432	0.00618	0.17647	-	-
TOG	0.01136	0.00618	0.01136	0.01136	0.07692	0.48022	0.07692	0.01136	-
UGA	1	0.00666	0.01136	0.01136	0.07692	0.00071	0.22857	0.01136	0.0035

PCB_trimer_3

	CAM	CON	GHA	IVC	MAD	SAT	SLE	TAN	TOG
CON	0.054	-	-	-	-	-	-	-	-
GHA	1	0.054	-	-	-	-	-	-	-
IVC	1	0.054	0.662	-	-	-	-	-	-
MAD	0.222	0.804	0.222	0.222	-	-	-	-	-
SAT	0.533	0.436	0.012	0.012	0.727	-	-	-	-
SLE	0.414	0.161	0.222	0.222	0.484	0.092	-	-	-
TAN	1	0.054	0.092	0.092	0.222	0.092	0.222	-	-
TOG	1	0.012	0.092	0.092	0.111	0.112	0.111	1	-
UGA	0.809	0.012	0.809	0.732	0.111	0.382	0.111	1	1

PCB_trimer_4

	CAM	CON	GHA	IVC	MAD	SAT	SLE	TAN	TOG
CON	0.95455	-	-	-	-	-	-	-	-
GHA	0.05143	0.02381	-	-	-	-	-	-	-
IVC	0.73469	0.02381	0.05143	-	-	-	-	-	-
MAD	0.16216	0.10714	0.16216	0.16216	-	-	-	-	-
SAT	0.00706	0.00162	0.00706	0.00706	0.61813	-	-	-	-
SLE	1	0.70557	0.16216	0.16216	0.38462	0.04945	-	-	-
TAN	0.05143	0.02381	0.05143	0.05143	0.16216	0.04685	0.16216	-	-
TOG	0.01299	0.00706	0.01299	0.01299	0.06897	0.00036	0.06897	0.01299	-
UGA	0.25359	0.00749	0.01299	0.95455	0.06897	0.00036	0.06897	0.01299	0.02098

PCB_trimer_5

	CAM	CON	GHA	IVC	MAD	SAT	SLE	TAN	TOG
CON	0.02381	-	-	-	-	-	-	-	-
GHA	0.05143	0.02381	-	-	-	-	-	-	-
IVC	0.05143	0.02381	0.05143	-	-	-	-	-	-
MAD	0.16216	0.11084	0.16216	0.16216	-	-	-	-	-
SAT	0.00549	0.00162	0.00549	0.00549	0.80919	-	-	-	-
SLE	0.16216	0.85714	0.16216	0.16216	0.38462	0.05143	-	-	-
TAN	0.05143	0.52265	0.05143	0.05143	0.16216	0.00549	0.16216	-	-
TOG	0.01212	0.27052	0.01212	0.01212	0.07143	0.00036	0.74419	0.61039	-
UGA	0.51818	0.00549	0.01212	0.01212	0.07143	0.00036	0.07143	0.01212	0.00175

PCA_pentoside_1

	CAM	CON	GHA	IVC	MAD	SAT	SLE	TAN	TOG
CON	0.5102	-	-	-	-	-	-	-	-
GHA	0.05357	0.02679	-	-	-	-	-	-	-
IVC	0.09184	0.02679	0.05357	-	-	-	-	-	-
MAD	0.15385	0.10557	0.15385	0.15385	-	-	-	-	-
SAT	0.00549	0.00162	0.00549	0.00549	0.05205	-	-	-	-
SLE	0.55814	0.10557	0.15385	0.15385	0.36585	0.05205	-	-	-
TAN	0.05357	0.05042	0.05357	0.05357	0.15385	0.00549	0.15385	-	-
TOG	0.24091	0.58578	0.01399	0.01399	0.07407	0.00036	0.07407	0.02597	-
UGA	0.15341	0.00549	0.01399	0.10557	0.07407	0.00036	0.71111	0.01399	0.00175

PCB_trimer_7

	CAM	CON	GHA	IVC	MAD	SAT	SLE	TAN	TOG
CON	0.214	-	-	-	-	-	-	-	-
GHA	1	0.071	-	-	-	-	-	-	-
IVC	1	0.071	0.125	-	-	-	-	-	-
MAD	0.214	0.169	0.214	0.214	-	-	-	-	-
SAT	0.154	0.422	0.045	0.125	0.651	-	-	-	-
SLE	0.214	0.169	0.214	0.214	0.469	0.651	-	-	-
TAN	0.791	0.125	0.125	0.125	0.214	0.487	0.214	-	-
TOG	0.576	0.47	0.045	0.045	0.125	0.357	0.125	0.487	-
UGA	0.332	0.935	0.045	1	0.125	0.08	0.125	1	0.898

PCA_trimer_arabinoside

	CAM	CON	GHA	IVC	MAD	SAT	SLE	TAN	TOG
CON	0.16667	-	-	-	-	-	-	-	-
GHA	1	0.02381	-	-	-	-	-	-	-
IVC	1	0.02381	0.0559	-	-	-	-	-	-
MAD	0.16667	0.11084	0.16667	0.16667	-	-	-	-	-
SAT	0.00549	0.00162	0.00549	0.00549	0.60306	-	-	-	-
SLE	1	0.11084	0.16667	0.6	0.40541	0.05205	-	-	-
TAN	0.0559	0.54945	0.0559	0.0559	0.16667	0.00549	0.16667	-	-
TOG	0.01136	0.40854	0.01136	0.01136	0.07407	0.00036	0.07407	0.01136	-
UGA	1	0.00549	0.01136	0.01136	0.07407	0.00036	0.07407	0.01136	0.00175

PCA_hexoside_2

	CAM	CON	GHA	IVC	MAD	SAT	SLE	TAN	TOG
CON	0.16667	-	-	-	-	-	-	-	-
GHA	0.0559	0.02679	-	-	-	-	-	-	-
IVC	0.09184	0.02679	0.0559	-	-	-	-	-	-
MAD	1	0.72321	0.16667	0.16667	-	-	-	-	-
SAT	0.00618	0.06	0.00618	0.00618	0.05495	-	-	-	-
SLE	1	0.72321	0.16667	0.16667	0.73171	0.05495	-	-	-
TAN	1	0.02679	0.0559	0.0559	0.16667	0.00618	0.16667	-	-
TOG	1	0.12608	0.01399	0.01399	0.47368	0.00036	0.21622	0.08081	-
UGA	0.01399	0.00618	0.0559	0.01399	0.07692	0.00036	0.07692	0.01399	0.00233

N-coumaroyl-L-tyrosine

	CAM	CON	GHA	IVC	MAD	SAT	SLE	TAN	TOG
CON	0.02521	-	-	-	-	-	-	-	-
GHA	0.10714	0.02521	-	-	-	-	-	-	-
IVC	1	0.02521	0.06429	-	-	-	-	-	-
MAD	0.1875	0.12363	0.1875	0.1875	-	-	-	-	-
SAT	0.00549	0.00162	0.00549	0.00549	0.33333	-	-	-	-
SLE	1	0.12363	0.1875	0.92308	0.40541	0.05495	-	-	-
TAN	1	0.02521	0.06429	0.1875	0.1875	0.00549	0.33333	-	-
TOG	1	0.00549	0.01399	0.80861	0.08696	0.00036	1	0.29201	-
UGA	1	0.00549	0.01399	0.01399	0.08696	0.00036	0.08696	0.01399	0.31037

PCA_pentoside_2

	CAM	CON	GHA	IVC	MAD	SAT	SLE	TAN	TOG
CON	0.18182	-	-	-	-	-	-	-	-
GHA	0.06767	0.03061	-	-	-	-	-	-	-
IVC	0.06767	0.03061	0.06767	-	-	-	-	-	-
MAD	1	0.68878	0.18182	0.18182	-	-	-	-	-
SAT	0.11395	0.15574	0.00824	0.00824	0.18182	-	-	-	-
SLE	1	0.68878	0.18182	0.18182	1	0.18182	-	-	-
TAN	0.44082	0.54945	0.06767	0.06767	0.18182	0.4739	0.18182	-	-
TOG	0.28342	0.09091	0.01515	0.54545	0.48649	0.00824	0.6	0.01515	-
UGA	0.01515	0.00824	0.01515	0.01515	0.09091	0.00071	0.09091	0.01515	0.0035

Quercetin-3-O-glucoside

	CAM	CON	GHA	IVC	MAD	SAT	SLE	TAN	TOG
CON	0.02679	-	-	-	-	-	-	-	-
GHA	0.05357	0.02679	-	-	-	-	-	-	-
IVC	0.71761	0.02679	0.05357	-	-	-	-	-	-
MAD	0.15385	0.10369	0.15385	0.15385	-	-	-	-	-
SAT	0.00618	0.06897	0.00618	0.00618	0.70644	-	-	-	-
SLE	1	0.10369	0.15385	0.15385	0.375	0.04945	-	-	-
TAN	0.15385	0.02679	0.05357	0.05357	0.15385	0.00618	0.15385	-	-
TOG	0.03828	0.00618	0.03828	0.03828	0.06897	0.00036	0.06897	0.01653	-
UGA	0.50554	0.00618	0.01653	0.95455	0.06897	0.00036	0.06897	0.01653	0.01748

Quercetin-3-O-arabinoside

	CAM	CON	GHA	IVC	MAD	SAT	SLE	TAN	TOG
CON	0.02381	-	-	-	-	-	-	-	-
GHA	0.0559	0.02381	-	-	-	-	-	-	-
IVC	0.92691	0.02381	0.0559	-	-	-	-	-	-
MAD	0.16667	0.70557	0.16667	0.16667	-	-	-	-	-
SAT	0.00618	0.16667	0.00618	0.00618	0.40575	-	-	-	-
SLE	1	0.1148	0.16667	0.16667	0.39474	0.05205	-	-	-
TAN	0.16667	0.02381	0.0559	0.0559	0.16667	0.01413	0.32432	-	-
TOG	0.8658	0.00599	0.01399	0.01399	0.07692	0.00036	0.6	0.08081	-
UGA	1	0.00599	0.01399	0.01399	0.07692	0.00036	0.07692	0.01399	0.00233

FDR (false discovery rate)<0.001; FDR<0.01 and FDR<0.05 are highlighted in green, yellow, and red, respectively. Compounds highlighted in bold were used to build the box plots that are displayed in the main manuscript.

Table S4. Pairwise comparison of the phenolic compounds quantified in CBSs by RP-HPLC-PDA that could be used as potential geographical origin markers for different countries within the American continent.

Protocatechuic_acid

	BRA	COL	DOR	ECU	JAM	MEX	PER
COL	0.00665	-	-	-	-	-	-
DOR	0.00203	0.00665	-	-	-	-	-
ECU	0.00062	0.26582	0.01404	-	-	-	-
JAM	0.03333	0.00665	0.0122	0.02765	-	-	-
MEX	0.03333	0.00665	0.00203	0.00062	0.03333	-	-
PER	0.00665	0.02765	0.00024	0.1995	0.00665	0.00665	-
VEN	0.00163	0.00665	2.10E-07	0.07636	0.00024	0.00024	0.77807

N-Coumaroyl-L-aspartate_2

	BRA	COL	DOR	ECU	JAM	MEX	PER
COL	0.01028	-	-	-	-	-	-
DOR	0.00261	0.01156	-	-	-	-	-
ECU	0.00075	0.53208	0.06623	-	-	-	-
JAM	0.05	0.01028	0.77637	0.06724	-	-	-
MEX	0.05	0.83801	0.05471	0.12208	0.05	-	-
PER	0.01028	0.19543	0.05471	0.05	0.01028	1	-
VEN	0.00032	0.11724	0.00037	0.38674	0.00032	0.00111	0.00025

PCA_pentoside_1

	BRA	COL	DOR	ECU	JAM	MEX	PER
COL	0.057	-	-	-	-	-	-
DOR	0.16	0.765	-	-	-	-	-
ECU	0.224	0.801	0.627	-	-	-	-
JAM	0.16	1	0.765	0.923	-	-	-
MEX	0.16	1	0.984	0.627	0.229	-	-
PER	0.057	0.765	0.308	0.976	1	0.255	-
VEN	0.627	0.627	0.303	0.627	0.765	0.529	0.765

N-Coumaroyl-L-aspartate_1

	BRA	COL	DOR	ECU	JAM	MEX	PER
COL	0.00943	-	-	-	-	-	-
DOR	0.00305	0.97338	-	-	-	-	-
ECU	0.00187	0.17333	0.04875	-	-	-	-
JAM	0.04875	0.00943	0.00305	0.0025	-	-	-
MEX	0.04875	0.00943	0.00305	0.18223	0.04875	-	-
PER	0.00943	0.19012	0.07803	0.19012	0.00943	0.00943	-
VEN	0.03766	0.92114	0.97338	0.19012	0.00095	0.19012	0.55508

PCB_trimer_2

	BRA	COL	DOR	ECU	JAM	MEX	PER
COL	1	-	-	-	-	-	-
DOR	0.06062	0.16207	-	-	-	-	-
ECU	0.50363	1	0.03645	-	-	-	-
JAM	0.05333	0.01131	0.00366	0.00795	-	-	-
MEX	0.05333	1	0.29614	0.22098	0.05333	-	-
PER	1	0.16207	0.00293	0.19538	0.01131	0.01131	-
VEN	0.00795	0.02473	0.69453	0.00197	0.00048	0.1067	0.00048

PCB_trimer_7

	BRA	COL	DOR	ECU	JAM	MEX	PER
COL	0.01187	-	-	-	-	-	-
DOR	0.01187	0.87813	-	-	-	-	-
ECU	0.46586	0.08768	0.04069	-	-	-	-
JAM	0.05	0.01886	0.05705	0.87813	-	-	-
MEX	0.05	0.01187	0.01187	0.00094	0.05	-	-
PER	1	0.01187	0.00904	0.7123	0.9679	0.01187	-
VEN	0.6304	0.00092	0.00092	0.72581	0.84894	0.00092	0.84894

N-Caffeoyl-L-aspartate

	BRA	COL	DOR	ECU	JAM	MEX	PER
COL	0.0087	-	-	-	-	-	-
DOR	0.00203	0.6903	-	-	-	-	-
ECU	0.00062	0.05827	0.06292	-	-	-	-
JAM	0.04706	1	0.85943	0.06404	-	-	-
MEX	0.04706	0.0087	0.00203	0.00062	0.04706	-	-
PER	0.0087	1	0.22838	0.01986	0.09256	0.0087	-
VEN	0.00032	0.05079	0.00089	0.63787	0.00032	0.00032	0.00059

PCB

	BRA	COL	DOR	ECU	JAM	MEX	PER
COL	0.01257	-	-	-	-	-	-
DOR	0.00458	0.51166	-	-	-	-	-
ECU	0.00187	0.42694	0.14592	-	-	-	-
JAM	0.05714	0.01257	0.00458	0.01257	-	-	-
MEX	0.05714	0.6903	0.77637	0.42694	0.05714	-	-
PER	0.01257	1	0.6903	0.6903	0.01257	1	-
VEN	0.00095	0.06671	0.03345	0.12914	0.02805	0.16992	0.14726

PCA_trimer_arabinoside

	BRA	COL	DOR	ECU	JAM	MEX	PER
COL	0.00943	-	-	-	-	-	-
DOR	0.00985	0.76843	-	-	-	-	-
ECU	0.10863	0.0251	0.07375	-	-	-	-
JAM	0.04211	0.93333	0.49485	0.45875	-	-	-
MEX	0.04211	0.00943	0.00432	0.00125	0.04211	-	-
PER	0.00943	0.00746	0.03439	0.45875	0.00943	0.00943	-
VEN	0.00095	0.00432	0.0251	0.38929	0.00602	0.00095	0.8334

Catechin

	BRA	COL	DOR	ECU	JAM	MEX	PER
COL	0.01886	-	-	-	-	-	-
DOR	0.0061	0.20529	-	-	-	-	-
ECU	0.00187	0.15817	0.72262	-	-	-	-
JAM	0.0834	1	0.37744	0.72262	-	-	-
MEX	0.0834	1	0.15817	0.37744	0.0834	-	-
PER	0.01886	0.37744	0.99113	0.72262	0.3527	0.01886	-
VEN	0.00095	0.0834	0.37744	0.79231	0.10236	0.10236	0.34485

Epicatechin

	BRA	COL	DOR	ECU	JAM	MEX	PER
COL	0.00808	-	-	-	-	-	-
DOR	0.00261	0.00925	-	-	-	-	-
ECU	0.00094	0.04	0.69001	-	-	-	-
JAM	0.04	0.00808	0.02059	0.00749	-	-	-
MEX	0.04	1	0.28326	0.00094	0.04	-	-
PER	0.00808	0.61787	0.13997	0.34754	0.00808	0.00808	-
VEN	0.00048	0.00129	0.12347	0.00598	0.50432	0.00048	0.00129

PCA_hexoside_2

	BRA	COL	DOR	ECU	JAM	MEX	PER
COL	0.01616	-	-	-	-	-	-
DOR	0.08818	0.6429	-	-	-	-	-
ECU	0.71907	0.00946	0.08905	-	-	-	-
JAM	0.08	0.01616	0.1958	0.65484	-	-	-
MEX	0.08	1	0.36195	0.0025	0.08	-	-
PER	1	0.1958	0.71907	0.44829	1	0.3527	-
VEN	1	3.30E-05	0.00681	0.38109	0.24848	0.00048	0.15059

PCA_hexoside_1

	BRA	COL	DOR	ECU	JAM	MEX	PER
COL	0.00754	-	-	-	-	-	-
DOR	0.00229	8.80E-05	-	-	-	-	-
ECU	0.00075	0.03211	0.97258	-	-	-	-
JAM	0.0381	0.00754	0.97258	0.97258	-	-	-
MEX	0.0381	0.00754	0.00229	0.00075	0.0381	-	-
PER	0.00754	0.27358	8.80E-05	0.02008	0.00754	0.00754	-
VEN	0.02008	0.0024	0.10165	0.05258	0.27726	0.00032	0.00108

N-Coumaroyl-L-glutamate

	BRA	COL	DOR	ECU	JAM	MEX	PER
COL	0.9333	-	-	-	-	-	-
DOR	0.0693	0.1478	-	-	-	-	-
ECU	0.2541	0.8241	0.0774	-	-	-	-
JAM	0.0667	0.0323	0.8074	0.0693	-	-	-
MEX	0.0667	0.0226	0.3258	0.0037	0.0667	-	-
PER	0.0226	0.0827	0.7966	0.0827	0.7966	0.0226	-
VEN	0.4204	0.8601	0.0428	0.596	0.0455	0.0307	0.0226

N-coumaroyl-L-tyrosine

	BRA	COL	DOR	ECU	JAM	MEX	PER
COL	0.0087	-	-	-	-	-	-
DOR	0.0087	0.00949	-	-	-	-	-
ECU	0.00075	0.55401	0.01738	-	-	-	-
JAM	0.04	0.0087	0.2606	0.00949	-	-	-
MEX	0.04	0.0087	0.2606	0.00075	0.04	-	-
PER	0.0087	0.30007	0.01114	0.2606	0.0087	0.0087	-
VEN	0.00032	0.2606	0.0087	0.72046	0.00032	0.00032	0.20455

Catechin-3-O-glucoside

	BRA	COL	DOR	ECU	JAM	MEX	PER
COL	0.93333	-	-	-	-	-	-
DOR	0.06062	0.08262	-	-	-	-	-
ECU	0.32061	0.24189	0.01049	-	-	-	-
JAM	0.05333	0.01049	0.31024	0.00095	-	-	-
MEX	0.05333	0.01049	0.00366	0.00095	0.05333	-	-
PER	0.93333	0.77636	0.01486	0.2504	0.01049	0.01049	-
VEN	0.32745	0.25957	0.01049	0.44396	0.00095	0.00095	0.34186

PCB_trimer_3

	BRA	COL	DOR	ECU	JAM	MEX	PER
COL	0.01616	-	-	-	-	-	-
DOR	0.01165	8.80E-05	-	-	-	-	-
ECU	0.53357	0.01616	0.03306	-	-	-	-
JAM	0.14545	0.31677	0.00261	0.23941	-	-	-
MEX	0.08421	0.0115	0.11072	0.4191	0.04444	-	-
PER	0.5697	0.00653	0.00042	0.29905	0.14545	0.01616	-
VEN	0.0115	2.90E-06	0.03513	0.00024	0.00111	0.02275	0.00024

PCA_pentoside_2

	BRA	COL	DOR	ECU	JAM	MEX	PER
COL	0.01004	-	-	-	-	-	-
DOR	0.18377	0.06688	-	-	-	-	-
ECU	0.15324	0.00121	0.01004	-	-	-	-
JAM	0.04444	0.01004	0.87843	0.02146	-	-	-
MEX	0.04444	0.01616	0.01004	0.01004	0.04444	-	-
PER	0.01004	0.01004	0.21011	0.00391	0.06688	0.01004	-
VEN	0.40767	0.00019	0.06677	0.27578	0.11771	0.00048	0.01004

Quercetin

	BRA	COL	DOR	ECU	JAM	MEX	PER
COL	0.01131	-	-	-	-	-	-
DOR	0.6695	0.37337	-	-	-	-	-
ECU	1	0.05255	0.43574	-	-	-	-
JAM	0.05714	0.46795	0.85943	0.24722	-	-	-
MEX	0.05714	0.01131	0.00366	0.00094	0.05714	-	-
PER	0.01131	0.34558	0.63934	0.09986	1	0.01131	-
VEN	0.72202	1.40E-05	0.09986	0.27939	0.00602	0.00032	5.00E-05

PCB_trimer_4

	BRA	COL	DOR	ECU	JAM	MEX	PER
COL	0.00808	-	-	-	-	-	-
DOR	0.00261	0.00053	-	-	-	-	-
ECU	0.00075	1.30E-05	0.37312	-	-	-	-
JAM	0.04211	0.00808	0.06497	0.00674	-	-	-
MEX	0.04211	0.12727	0.07479	0.07338	0.04211	-	-
PER	0.00808	0.06651	0.00285	0.00209	0.00808	0.30458	-
VEN	0.01753	7.30E-07	0.13302	0.01696	0.61007	0.00285	2.50E-06

Quercetin-3-O-glucoside

	BRA	COL	DOR	ECU	JAM	MEX	PER
COL	0.01257	-	-	-	-	-	-
DOR	0.06062	0.49407	-	-	-	-	-
ECU	0.00145	0.02897	0.08856	-	-	-	-
JAM	0.05333	0.34431	0.54409	0.0791	-	-	-
MEX	0.05333	0.34431	0.59511	0.12006	0.88571	-	-
PER	0.01257	0.00145	0.01387	0.37358	0.01257	0.01257	-
VEN	0.00285	0.00096	0.00366	0.18373	0.01677	0.02844	0.16417

PCB_trimer_1

	BRA	COL	DOR	ECU	JAM	MEX	PER
COL	1	-	-	-	-	-	-
DOR	0.61856	0.99113	-	-	-	-	-
ECU	0.5183	0.34514	0.32418	-	-	-	-
JAM	0.06154	0.01131	0.00305	0.00094	-	-	-
MEX	0.06154	0.01131	0.00305	0.00094	0.06154	-	-
PER	0.7596	1	0.32418	0.32418	0.01131	0.01131	-
VEN	0.92117	0.34514	0.88715	0.95717	0.00048	0.00048	0.91638

PCB_trimer_5

	BRA	COL	DOR	ECU	JAM	MEX	PER
COL	0.0132	-	-	-	-	-	-
DOR	1	0.0139	-	-	-	-	-
ECU	0.1844	0.0015	0.0362	-	-	-	-
JAM	0.0571	0.0132	0.0046	0.3603	-	-	-
MEX	0.0571	1	0.3258	0.0015	0.0571	-	-
PER	0.1273	0.0015	0.1969	0.3603	0.0132	0.0132	-
VEN	0.8427	0.0132	0.2646	0.4476	0.634	0.1209	0.9492

Quercetin-3-O-arabinoside

	BRA	COL	DOR	ECU	JAM	MEX	PER
COL	0.34431	-	-	-	-	-	-
DOR	0.06466	0.63917	-	-	-	-	-
ECU	0.43586	0.08272	0.1605	-	-	-	-
JAM	0.05714	0.20733	0.29614	0.08272	-	-	-
MEX	0.05714	0.01131	0.00366	0.00366	0.05714	-	-
PER	0.68283	0.38291	0.02068	0.20733	0.01131	0.01131	-
VEN	0.08889	0.00366	0.00666	0.45917	0.00366	0.00095	0.01131

PCB_trimer_6

	BRA	COL	DOR	ECU	JAM	MEX	PER
COL	0.01028	-	-	-	-	-	-
DOR	0.00366	0.27795	-	-	-	-	-
ECU	0.07819	0.02587	0.00576	-	-	-	-
JAM	0.04706	0.01028	0.10488	0.86502	-	-	-
MEX	0.04706	0.01028	0.00366	0.61052	0.04706	-	-
PER	0.01028	1	0.27795	0.08913	0.9679	0.01028	-
VEN	0.08421	1.50E-06	2.10E-07	0.02673	0.01909	0.10488	0.00051

FDR (false discovery rate)<0.001; FDR<0.01 and FDR<0.05 are highlighted in green, yellow, and red, respectively. Compounds highlighted in bold were used to build the box plots that are displayed in the main manuscript.

Table S5. Pairwise comparison of the phenolic compounds quantified in CBSs by RP-HPLC-PDA that could be used as potential markers for cultivars among Ecuadorian samples.

Protocatechuic_acid

	Criollo	Forastero	Nacional
Forastero	0.79	-	-
Nacional	0.57	0.57	-
Trinitario	0.57	0.57	0.89

N-Coumaroyl-L-aspartate_2

	Criollo	Forastero	Nacional
Forastero	0.66	-	-
Nacional	0.2	1	-
Trinitario	0.2	0.2	0.17

PCA_pentoside_1

	Criollo	Forastero	Nacional
Forastero	0.16	-	-
Nacional	0.16	1	-
Trinitario	0.16	0.0066	0.0857

N-Coumaroyl-L-aspartate_1

	Criollo	Forastero	Nacional
Forastero	0.13	-	-
Nacional	0.27	0.95	-
Trinitario	0.27	0.57	0.58

PCB_trimer_2

	Criollo	Forastero	Nacional
Forastero	0.044	-	-
Nacional	0.1203	0.013	-
Trinitario	0.1333	0.0066	0.0441

PCB_trimer_7

	Criollo	Forastero	Nacional
Forastero	0.6593	-	-
Nacional	0.2	0.0033	-
Trinitario	0.2	0.0033	1

N-Caffeoyl-L-aspartate

	Criollo	Forastero	Nacional
Forastero	0.5495	-	-
Nacional	0.16	0.1165	-
Trinitario	0.16	0.0066	0.0857

PCB

	Criollo	Forastero	Nacional
Forastero	0.044	-	-
Nacional	0.16	0.0033	-
Trinitario	0.16	0.0033	1

PCA_trimer_arabinoside

	Criollo	Forastero	Nacional
Forastero	0.82	-	-
Nacional	0.96	0.76	-
Trinitario	0.64	0.64	1

Catechin

	Criollo	Forastero	Nacional
Forastero	0.13	-	-
Nacional	0.16	0.16	-
Trinitario	0.16	0.16	0.49

Epicatechin

	Criollo	Forastero	Nacional
Forastero	0.8242	-	-
Nacional	1	1	-
Trinitario	0.2667	0.0066	0.0857

PCA_hexoside_2

	Criollo	Forastero	Nacional
Forastero	0.16	-	-
Nacional	0.16	0.5209	-
Trinitario	0.16	0.0066	0.0857

PCA_hexoside_1

	Criollo	Forastero	Nacional
Forastero	0.5495	-	-
Nacional	0.2	0.4549	-
Trinitario	0.2	0.0066	0.0857

N-Coumaroyl-L-glutamate

	Criollo	Forastero	Nacional
Forastero	0.5275	-	-
Nacional	0.8	0.1165	-
Trinitario	0.2	0.0066	0.0857

N-coumaroyl-L-tyrosine

	Criollo	Forastero	Nacional
Forastero	0.8	-	-
Nacional	0.8	0.8	-
Trinitario	0.2667	0.0066	0.0857

Catechin-3-O-glucoside

	Criollo	Forastero	Nacional
Forastero	0.6593	-	-
Nacional	0.2	0.9527	-
Trinitario	0.2	0.0066	0.0857

PCB_trimer_3

	Criollo	Forastero	Nacional
Forastero	0.0659	-	-
Nacional	1	0.5687	-
Trinitario	0.2667	0.0066	1

PCA_pentoside_2

	Criollo	Forastero	Nacional
Forastero	0.044	-	-
Nacional	0.133	0.124	-
Trinitario	0.083	0.025	0.044

Quercetin

	Criollo	Forastero	Nacional
Forastero	0.64	-	-
Nacional	0.64	0.95	-
Trinitario	0.64	0.64	0.64

PCB_trimer_4

	Criollo	Forastero	Nacional
Forastero	0.659	-	-
Nacional	0.2	0.204	-
Trinitario	0.2	0.026	0.086

Quercetin-3-O-glucoside

	Criollo	Forastero	Nacional
Forastero	0.6593	-	-
Nacional	0.6593	0.8615	-
Trinitario	0.2667	0.0066	0.0857

PCB_trimer_1

	Criollo	Forastero	Nacional
Forastero	0.5495	-	-
Nacional	0.2	0.4549	-
Trinitario	0.2	0.0066	0.0857

PCB_trimer_5

	Criollo	Forastero	Nacional
Forastero	0.066	-	-
Nacional	1	0.066	-
Trinitario	0.2	0.156	0.583

Quercetin-3-O-arabinoside

	Criollo	Forastero	Nacional
Forastero	0.5495	-	-
Nacional	0.2	0.5495	-
Trinitario	0.2	0.0066	0.0857

PCB_trimer_6

	Criollo	Forastero	Nacional
Forastero	0.033	-	-
Nacional	0.1333	0.0066	-
Trinitario	0.0663	0.0123	0.033

FDR (false discovery rate)<0.001; FDR<0.01 and FDR<0.05 are highlighted in green, yellow, and red, respectively.
Compounds highlighted in bold were used to build the box plots that are displayed in the main manuscript.
